# HSV-1-induced disruption of transcription termination resembles a cellular stress response but selectively increases chromatin accessibility downstream of genes

**DOI:** 10.1371/journal.ppat.1006954

**Published:** 2018-03-26

**Authors:** Thomas Hennig, Marco Michalski, Andrzej J. Rutkowski, Lara Djakovic, Adam W. Whisnant, Marie-Sophie Friedl, Bhaskar Anand Jha, Marisa A. P. Baptista, Anne L’Hernault, Florian Erhard, Lars Dölken, Caroline C. Friedel

**Affiliations:** 1 Institut für Virologie, Julius-Maximilians-Universität Würzburg, Würzburg, Germany; 2 The Babraham Institute, Cambridge, United Kingdom; 3 Division of Infectious Diseases, Department of Medicine, University of Cambridge, Cambridge, United Kingdom; 4 Institut für Informatik, Ludwig-Maximilians-Universität München, München, Germany; University of Wisconsin-Madison, UNITED STATES

## Abstract

Lytic herpes simplex virus 1 (HSV-1) infection triggers disruption of transcription termination (DoTT) of most cellular genes, resulting in extensive intergenic transcription. Similarly, cellular stress responses lead to gene-specific transcription downstream of genes (DoG). In this study, we performed a detailed comparison of DoTT/DoG transcription between HSV-1 infection, salt and heat stress in primary human fibroblasts using 4sU-seq and ATAC-seq. Although DoTT at late times of HSV-1 infection was substantially more prominent than DoG transcription in salt and heat stress, poly(A) read-through due to DoTT/DoG transcription and affected genes were significantly correlated between all three conditions, in particular at earlier times of infection. We speculate that HSV-1 either directly usurps a cellular stress response or disrupts the transcription termination machinery in other ways but with similar consequences. In contrast to previous reports, we found that inhibition of Ca^2+^ signaling by BAPTA-AM did not specifically inhibit DoG transcription but globally impaired transcription. Most importantly, HSV-1-induced DoTT, but not stress-induced DoG transcription, was accompanied by a strong increase in open chromatin downstream of the affected poly(A) sites. In its extent and kinetics, downstream open chromatin essentially matched the poly(A) read-through transcription. We show that this does not cause but rather requires DoTT as well as high levels of transcription into the genomic regions downstream of genes. This raises intriguing new questions regarding the role of histone repositioning in the wake of RNA Polymerase II passage downstream of impaired poly(A) site recognition.

## Author summary

Recently, we reported that productive herpes simplex virus 1 (HSV-1) infection leads to disruption of transcription termination (DoTT) of most but not all cellular genes. This results in extensive transcription beyond poly(A) sites and into downstream genes. Subsequently, cellular stress responses were found to trigger transcription downstream of genes (DoG) for >10% of protein-coding genes. Here, we directly compared the two phenomena in HSV-1 infection, salt and heat stress and observed significant overlaps between the affected genes. We speculate that HSV-1 either directly usurps a cellular stress response or disrupts the transcription termination machinery in other ways with similar consequences. In addition, we show that inhibition of calcium signaling does not specifically inhibit stress-induced DoG transcription but globally impairs RNA polymerase I, II and III transcription. Finally, HSV-1-induced DoTT, but not stress-induced DoG transcription, was accompanied by a strong increase in chromatin accessibility downstream of affected poly(A) sites. In its kinetics and extent, this essentially matched poly(A) read-through transcription but does not cause but rather requires DoTT. We hypothesize that this results from impaired histone repositioning when RNA Polymerase II enters downstream intergenic regions of genes affected by DoTT.

## Introduction

Transcription termination is an essential process in gene expression that is coupled to all parts of RNA metabolism including transcription initiation, splicing, nuclear export and translation (reviewed in [1, 2]). It results in the release of RNA polymerase II (Pol II) and the nascent transcript from the chromatin, determines the general fate of individual transcripts and plays a crucial role in limiting the extent of pervasive transcription of the genome. Herpes simplex virus 1 (HSV-1) efficiently modulates cellular RNA metabolism and both cellular and viral gene expression to facilitate lytic infection [3–9]. Using 4-thiouridine-(4sU)-tagging followed by sequencing (4sU-seq), we recently reported that lytic HSV-1 infection results in the disruption of transcription termination (DoTT) of the majority but not all cellular genes [10]. This was dependent on *de novo* protein synthesis and already became broadly detectable by 2-3h of infection, which is before the release of the first newly generated virus particles at around 4h post infection (p.i.). At 7-8h p.i., about 50% of all 4sU-seq sequencing reads mapping to the human genome originated from intergenic regions (compared to <10% in uninfected cells). Previously, we referred to transcription beyond poly(A) sites due to DoTT as ‘read-out’. As this term has led to confusion, we now use the term ‘read-through’ to refer to transcription that extends beyond poly(A) sites. Transcription into a downstream gene arising from read-through from an upstream gene is referred to as ‘read-in’. For more than half of expressed cellular genes, poly(A) read-through affected >35% of their transcription. Read-in transcription into downstream genes was responsible for the seeming induction of about 1,100 cellular protein-coding and non-coding genes late in infection. In addition, it resulted in chimeric transcripts spanning two or more genes as evidenced by intergenic splicing events that connect exons of neighboring cellular genes.

Subsequently, two other studies reported on the disruption of transcription termination in cellular stress responses and cancer [11, 12]. Transcription downstream of genes (DoG) was observed in the osmotic stress response in human neuroblastoma cells, which was independent of *de novo* protein synthesis but appeared to at least partially rely on inositol-1,4,5-trisphosphate receptor (IP3R) activation and calcium signaling [11]. In addition, pervasive transcription read-through was identified in renal cell carcinoma [12]. This was dependent on the loss of histone methyltransferase SETD2, consistent with the role of epigenetic factors in RNA processing. Similar to HSV-1 infection, novel RNA chimeras were observed. Invasion of oncogenes by polymerases that initiated at upstream genes indicated a novel link between aberrant expression of oncogenes and chimeric transcripts prevalent in cancer. Taken together, these findings raise important questions regarding the underlying molecular mechanisms and functional roles of DoTT/DoG transcription in HSV-1 infection, cellular stress responses and cancer.

**Fig 1:**
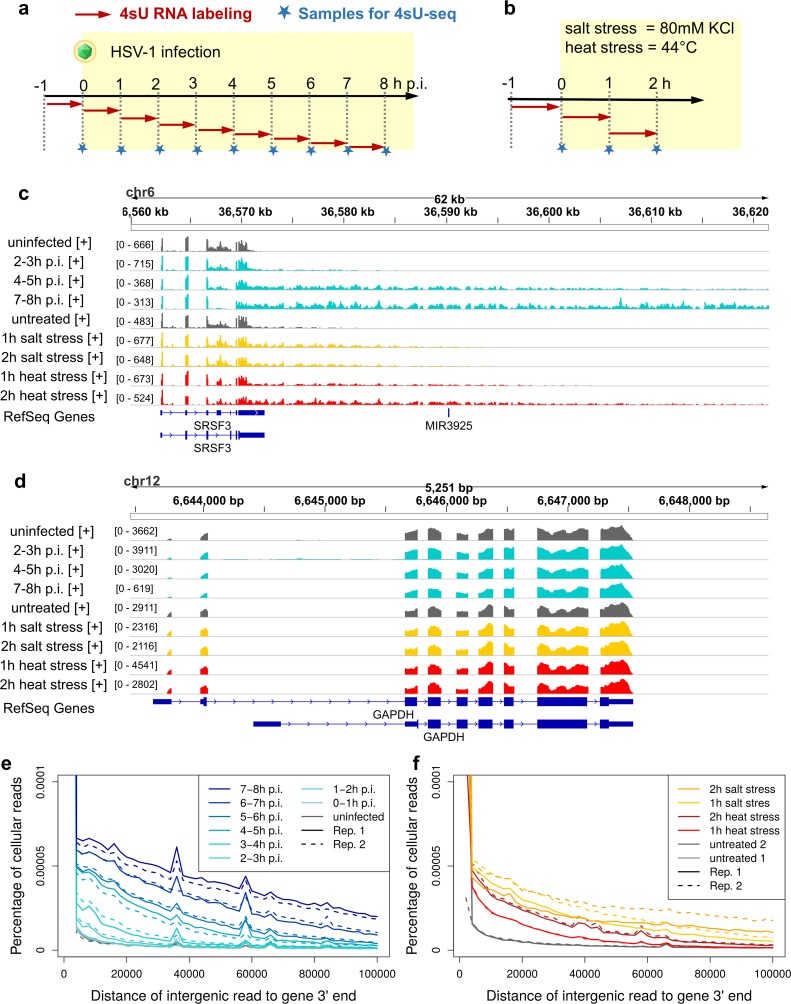
Experimental set-up and read distribution downstream of genes. (a-b) Experimental set-up of our original 4sU-seq time-course for HSV-1 infection [10] (a) and for the analysis of DoG transcription in salt and heat stress (b). 4sU-tagging was performed in 1h intervals before infection and stress as well as for the first 8h of HSV-1 infection and for the first 2h of salt and heat stress. Two biological 4sU-RNA replicates of each condition were subjected to Illumina sequencing (4sU-seq). (c-d) 4sU-seq read coverage (= number of mapped sequencing reads, sum of 2 replicates) for the genes SRSF3 (c) and GAPDH (d) in uninfected/untreated samples (gray), during HSV-1 infection (cyan) and in salt (yellow) and heat (red) stress. Read coverage ranges are indicated in square brackets on the y-axis. Only reads mapping to the corresponding strand are shown. RefSeq gene annotation is indicated below (blue). Boxes indicate coding regions and untranslated regions (UTRs; narrow boxes) and lines intronic regions. The transcribed strand is indicated by the direction of the arrowheads. (e-f) Distribution of reads mapping in sense direction downstream of annotated gene 3’ ends in HSV-1 infection (e) and salt and heat stress (f) (shown separately for the two replicates: solid lines = replicate 1, dashed lines = replicate 2). Only gene 3’ ends with no gene on either strand within the 100kb downstream region were considered. Read counts in sense direction to the gene were determined in 2kb windows downstream of gene 3’ ends and divided by window length and the total number of mapped reads. Reads counts mapping to the antisense strand are shown in Fig B in S3 File.

DoG transcription during osmotic stress was identified by Vilborg et al. upon exposure to 80mM KCl for 1h (from now on referred to as ‘salt stress’) in a human neuroblastoma cell line (SK-N-BE(2)C) by RNA-seq on nuclear, RiboMinus-treated RNA [11]. This revealed about 2,000 human genes to be affected. In addition, DoG transcription was also observed following heat stress (44°C) [11]. Recently, Vilborg et al. also reported on DoG transcription upon oxidative stress and found significant similarities but also clear stress-specific differences between the three stressors [13]. In our primary study, we analyzed newly transcribed RNA purified using 4sU-seq in one hour intervals of the first 8h of lytic HSV-1 infection of primary human foreskin fibroblasts (HFF) (Fig 1A). Under these conditions, the HSV-1 infected cells only start to lyse around 16 to 24h of infection. This allowed us to directly assess and quantify the relative frequency of transcripts experiencing DoTT as well as the extent of read-through transcription occurring within one hour intervals during the first eight hours of infection [10]. Throughout this manuscript, we refer to HSV-1-induced disruption of transcription termination as ‘DoTT’ to differentiate it from stress-induced DoG transcription. It is important to note here that transcription in intergenic regions downstream of genes was almost exclusively observed on the sense strand in relation to the upstream gene. This clearly distinguishes read-through from the recently reported activation of antisense transcription of the host genome during lytic HSV-1 infection [14].

Although DoTT was much more prominent at late times (7-8h p.i.) of HSV-1 infection than in salt or heat stress, we wondered whether the two phenomena might reflect the same cellular mechanism. We thus performed a detailed comparison and characterization of HSV-1-induced DoTT and DoG transcription triggered by salt and heat stress using 4sU-seq in the same cell type, namely HFF. This showed clear similarities in read-through between HSV-1 infection and the different stresses but also clear context- and condition-specific differences. Furthermore, we performed ATAC-seq (transposase-accessible chromatin using sequencing [15]) to compare chromatin accessibility before and during HSV-1 infection and stress. Strikingly, HSV-1-induced DoTT was accompanied by a strong increase in chromatin accessibility downstream of the affected poly(A) sites, which essentially matched the region of read-through transcription. This did not cause but rather required DoTT as well as a high level of transcriptional activity into downstream genomic regions. Interestingly, this effect was specific to HSV-1 and not observed in salt or heat stress (up to 2h) indicating that other mechanisms by which HSV-1 perturbs RNA processing contribute to this unexpected gene-specific alteration in the host chromatin landscape.

## Results

### Quantitative analysis of DoTT/DoG transcription in salt and heat stress

To directly compare HSV-1-induced DoTT with DoG transcription during cellular stress responses, we performed 4sU-seq analysis (60min 4sU-tagging followed by RNA sequencing) of HFF exposed to either salt (80mM KCl) or heat stress (44°C) for 1 and 2h (see Fig 1B). Two biological replicates of each condition as well as 2 untreated samples for each stressor were analyzed. 4sU-seq data for the first 8h of HSV-1 infection in HFF were obtained from our previous study [10]. A visual inspection of mapped reads for marker genes with either strong (SRSF3, SRSF6) or no (GAPDH, ACTB) DoTT/DoG transcription already indicated a striking similarity between presence or absence of DoTT/DoG transcription in the three conditions (Fig 1C and 1D; Fig A in S3 File, links to UCSC genome browser sessions showing read coverage for all cellular genes and samples separately for both replicates can be found at www.bio.ifi.lmu.de/HSV-1). As previously reported for HSV-1 infection [10] (Fig 1E), the percentage of reads mapping to intergenic regions downstream of gene 3’ ends increased substantially during salt and heat stress in HFF (Fig 1F). Intergenic read counts were highest directly downstream of gene 3’ ends and gradually decreased with increasing distance to gene 3’ ends. Furthermore, downstream intergenic transcription occurred almost exclusively in the same orientation as the upstream gene in all conditions (Fig B in S3 File). The low levels of antisense reads downstream of genes increased with increasing distance from gene 3’ ends as a consequence of read-through transcription for genes expressed from the opposite DNA strand outside of the 100kb downstream window considered. The gradual decrease in read levels downstream of genes was not due to differences in the length of read-through between genes, but was also observed at the level of individual genes (Fig B in S3 File and Fig C in S3 File). It could be approximated reasonably well by a linear fit at least late in HSV-1 infection and at 2h salt and heat stress, but the slope of the linear fit differed between genes (Fig C in S3 File). As a consequence of this gradual decrease and in contrast to regular mRNAs, 3’ ends of poly(A) read-through transcripts are not clearly defined [10, 11]. As the extent of read-through for individual genes gradually increased throughout infection, read-through transcripts extended further and further downstream of the gene.

To compare the extent of DoTT/DoG transcription between the three conditions, we focused on the 9,404 protein-coding and lincRNA (long intergenic non-coding RNA) genes whose expression was well detectable (fragments per kilobase of exons per million mapped reads (FPKM) ≥1) in all uninfected/untreated 4sU-seq samples. We then applied our previous approach [10] of dividing expression in the 5kb downstream of genes by the gene expression (FPKM) value (see methods). This measure (denoted as percentage of downstream transcription) is independent of any normalization to sequencing depth, which is canceled out in the division. As 4sU-tagging provides newly transcribed RNA from defined intervals of infection and stress, the obtained ratios quantify the percentage of transcripts newly transcribed in this interval that experience poly(A) read-through. To avoid confounding effects due to transcription of neighboring genes, we only included genes separated from neighboring genes on the same strand by at least 5kb on either side (5,928 genes). Although the restriction to the first 5kb downstream of a gene is relatively arbitrary, using a larger window of e.g. 10kb resulted in highly correlated values of downstream transcription (Spearman correlation $R_{s}>0.95$) but would exclude an additional 737 genes (12.4%) from the analysis. To account for small levels of downstream transcription in uninfected and untreated cells (mean = 4.2% and 0.06%, respectively), we calculated read-through as the difference in the percentage of downstream transcription between infected/stressed and uninfected/untreated samples (see methods). Read-in was quantified in the same way by first quantifying transcription in the 5kb upstream of genes relative to gene expression and then subtracting levels in uninfected/untreated samples. Since our previous study indicated that genes with read-in were more prone to read-through, we only used genes for the comparative analysis with at most 10% read-in in both HSV-1 infection and salt and heat stress (3,682 genes, Table A in S1 File). With the exception of the first three hours of HSV-1 infection where DoTT was hardly detectable, read-through values were highly correlated between replicates (Fig D in S3 File; $R_{s}\geq 0.85$).

**Fig 2:**
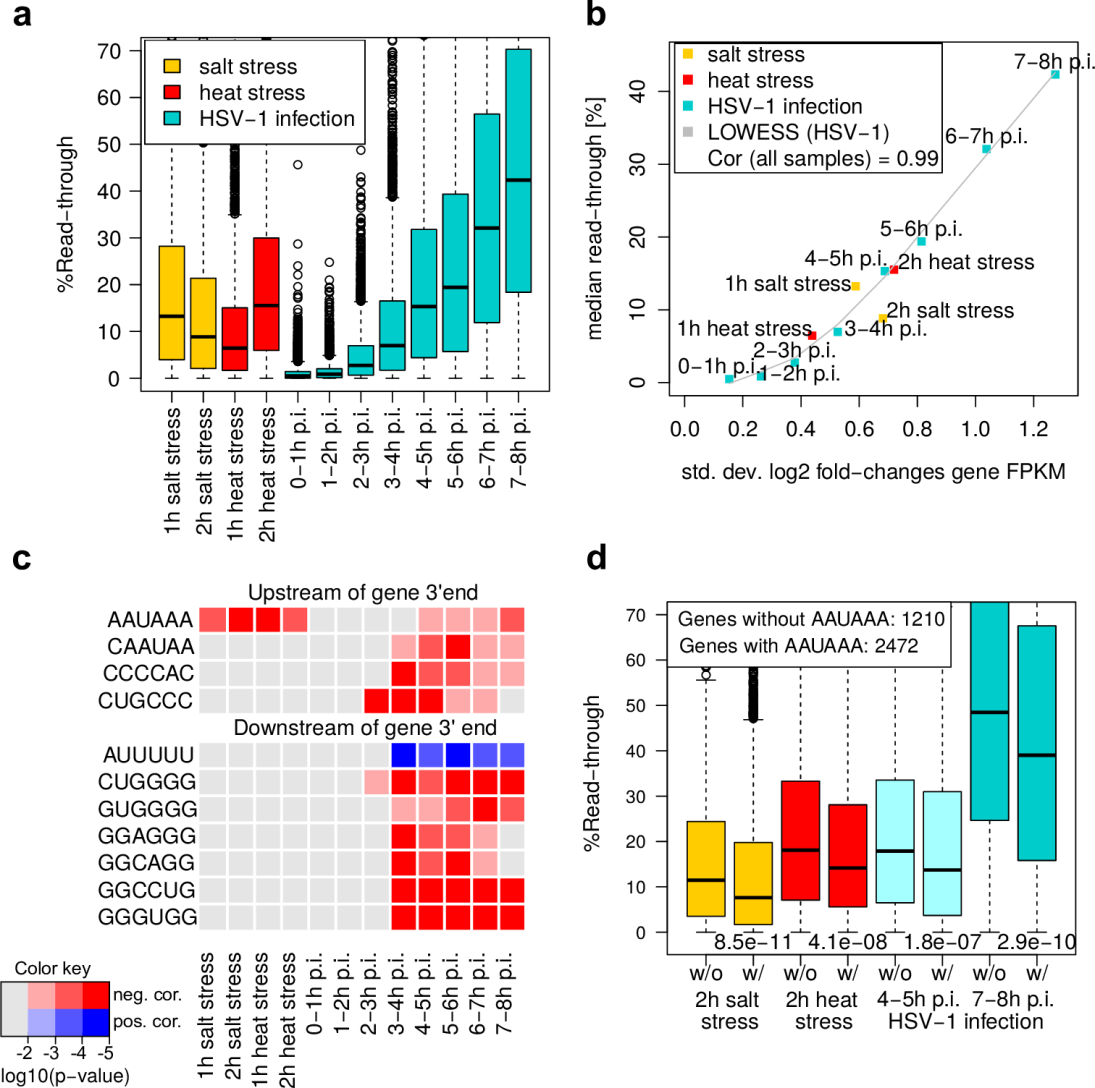
Global characteristics of DoTT/DoG transcription in salt and heat stress and HSV-1 infection. (a) Boxplots showing the distribution of read-through in salt (orange) and heat stress (red) and HSV-1 infection (cyan). Results for individual replicates are shown in Fig D in S3 File. (b) Median read-through values for each condition and time-point are plotted against the standard deviation in gene expression (= gene FPKM) fold-changes (log2). The gray curve indicates the result of a locally weighted polynomial regression (LOWESS) on all HSV-1 infection time-points. Spearman correlation (Cor) between median read-through and standard deviation in log2 expression fold-changes across all samples is also indicated. (c) 6-mers whose frequency in the 100nt up- or downstream of gene 3’ ends is significantly correlated to read-through in at least one sample (FDR adjusted p<0.0001). FDR adjusted p-values for all samples are color-coded (red for negative correlations, blue for positive correlations). (d) Boxplots showing the distribution of read-through in 2h salt and heat stress and 4-5h and 7-8h p.i. for genes without (w/o) or with (w/) at least one occurrence of the AAUAAA motif in the 100nt upstream of gene 3’ends. P-values of Wilcoxon rank sum tests comparing read-through in each sample between the two groups are indicated above the x-axis.

The induction of DoG transcription upon salt and heat stress was reflected in median read-through levels of 6 to 15% (Fig 2A; Fig D in S3 File for individual replicates). Consistent with the recent report by Vilborg et al. [13], global read-through levels peaked at 1h of salt stress, but required 2h to reach comparable levels in heat stress. At the highest level, read-through in both salt and heat stress was comparable to read-through at 4-5h post HSV-1 infection, but considerably lower (~3-fold) than at the end of our HSV-1 infection time-course (7-8h p.i.). Median read-through levels in all conditions were highly correlated ($R_{s}\;=\;0.99$) to the overall perturbation of gene expression (measured as standard deviation of FPKM log2 fold-changes; Fig 2B). Here, results for salt and heat stress fitted very well to a curve estimated from our HSV-1 time-course. At single gene level, however, read-through showed only a weak positive correlation with fold-changes in gene expression for HSV-1 infection (after the first 3h), salt and heat stress (Fig E in S3 File; $R_{s}\leq$ 0.37). Vilborg et al. [13] also only found weak correlations between fold-changes in DoG transcription and fold-changes in expression of the respective genes ($R_{s}\;=\;0.12$). The even lower correlations observed by Vilborg et al. may be explained by their use of nuclear RNA, which also contains RNA produced before stress. This underestimates gene expression changes for genes with low basal RNA turnover [16]. It should be noted that gene expression fold-changes estimated from RNA-seq data (even after normalization to house-keeping genes as performed here) only indicate changes in the relative, but not absolute, abundance among all expressed genes. As the overall transcription levels decline during lytic HSV-1 infection [17], positive fold-changes do not necessarily indicate actual transcriptional induction but only less down-regulation compared to other genes.

### RNA motifs associated with DoTT/DoG transcription

In our previous study, we reported that DoTT-induced read-through was increased for genes without the canonical AAUAAA poly(A)-signal upstream of the gene 3’end. Similarly, Vilborg et al. found several 6-mers to be depleted (including AAUAAA) or enriched downstream of genes with pan-stress DoG transcription. However, their analysis focused on the total frequency of the 6-mers downstream of all pan-stress DoG genes instead of the frequency for individual genes. We now aimed to identify 6-mers whose abundance in the 100nt up- or downstream of individual gene 3’ends was significantly correlated to read-through (FDR adjusted p-value <0.0001 for at least one condition or time-point, see methods). Strikingly, AAUAAA was the only 6-mer whose abundance upstream of gene 3’ends was significantly correlated with read-through in both stresses and HSV-1 infection (Fig 2C) and its absence upstream of gene 3’ ends was associated with significantly higher read-through (Wilcoxon rank sum test, p<0.0001; Fig 2D). Other 6-mers were only significantly correlated to read-through in HSV-1 infection and showed no significant differences in read-through in salt or heat stress (Fig F in S3 File). Upstream of gene 3’ends, negative correlations were found for a 6-mer overlapping the AAUAAA sequence as well as two C-rich motifs. Downstream of gene 3’ends, this included a number of G-rich motifs. Only one motif downstream of genes was positively correlated to read-through (AUUUUU), but only in HSV-1 infection. This sequence resembles binding motifs of a number of RNA binding proteins [18, 19], including HNRNPC (Heterogeneous Nuclear Ribonucleoprotein C), which has been shown to influence poly(A) site usage.

### Similarities and differences between DoTT and DoG transcription

**Fig 3:**
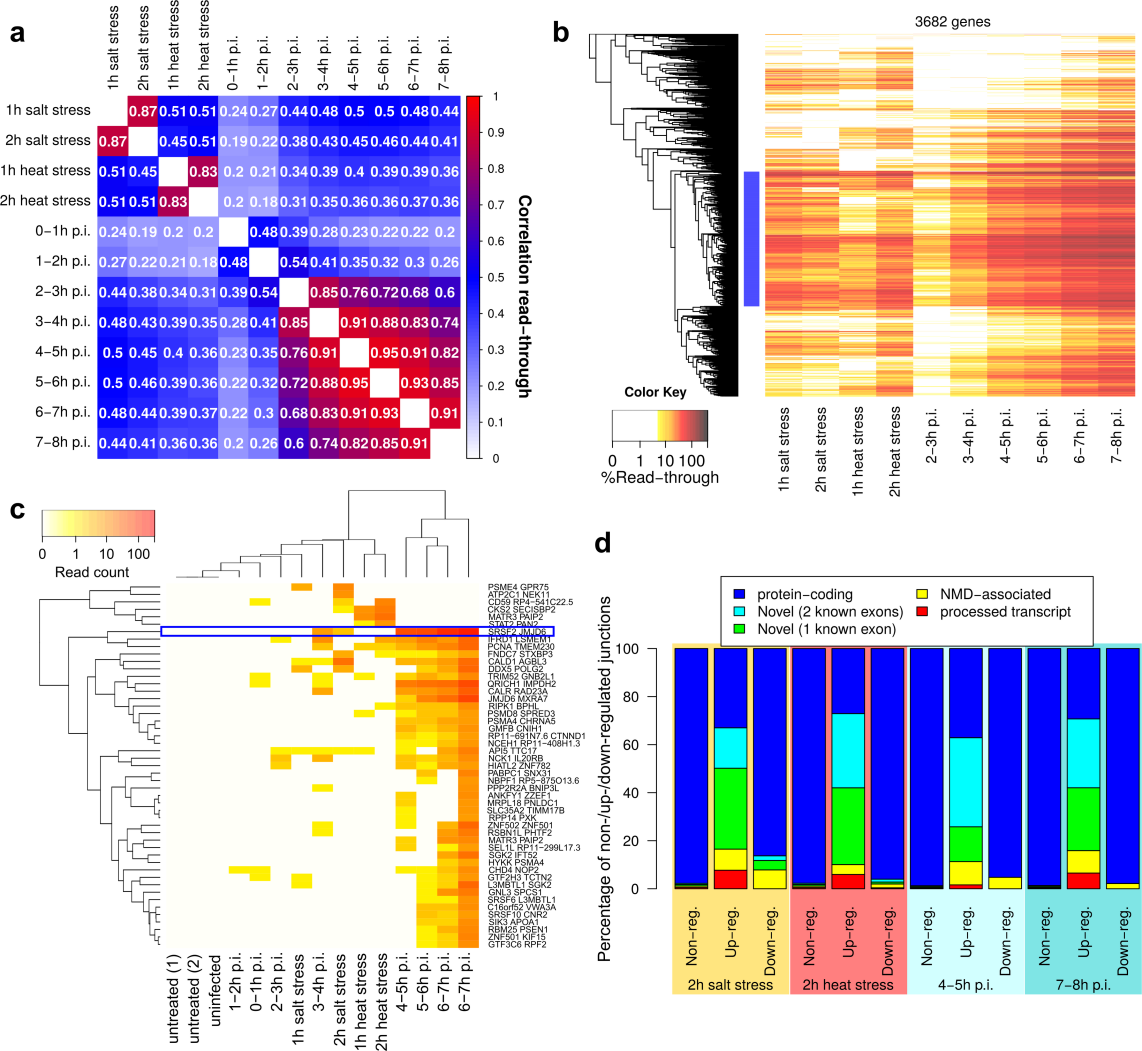
Comparison of DoTT/DoG transcription and association with aberrant splicing. (a) Spearman correlation for read-through values between all samples. (b) Heatmap of read-though for all 3,682 analyzed genes in salt and heat stress and HSV-1 infection (excluding the first two time-points with very low levels of read-through). Colors indicate read-though >5%. Hierarchical clustering was performed using average linkage clustering based on Euclidean distances. (c) Heatmap of read counts (sum of 2 replicates) for intergenic splicing events connecting exons of neighboring genes on the same strand. Junctions are annotated with the upstream and downstream gene symbol. Results for the intergenic splicing junction connecting SRSF2 and JMJD6 are highlighted by a blue box. Only junctions are shown with >2 reads covering at least 5bp of both exons in either 2h salt stress, 2h heat stress or 7-8h p.i. HSV-1 infection. Hierarchical clustering was performed as for (b). (d) Percentage of splicing junctions that are part of protein-coding transcripts, novel (using either 2 or 1 known exon boundary), nonsense-mediated-decay (NMD)-associated or only observed in a processed transcript. Results are shown separately for non-regulated, up-regulated and down-regulated junctions (see methods for definition) for 2h salt and heat stress and 4–5hs and 7-8h p.i. HSV-1 infection.

To directly compare HSV-1-induced DoTT to DoG transcription, we calculated Spearman rank correlations of read-through values between each pair of conditions and time-points. This compares the ranking of genes with regard to read-through, i.e. whether top- and lowest-ranked genes tend to be the same between samples. Read-through mostly correlated extremely well ($R_{s}>0.8$) between adjacent time-points for the same condition apart from the first three hours of HSV-1 infection where DoTT was hardly noticeable (Fig 3A). Moderate but comparable correlations were observed between salt stress and either heat stress or HSV-1 infection at 4-5h p.i. ($R_{s}\;=\;0.45-0.51$). In contrast, read-through in heat stress was slightly better correlated to salt stress than to HSV-1 infection ($R_{s}\;=\;0.4$). Since we observed a weak correlation between read-through and gene expression fold-changes in all conditions, we also calculated correlations after excluding genes with highest fold-changes (≥2 in any sample). This aimed to exclude genes for which differences in read-through between conditions might be explained by changes in transcriptional activity. However, correlations for the remaining 2,601 genes did not increase, which is probably explained by the observation that gene expression fold-changes were also well correlated (Fig G in S3 File). Thus, differences between conditions in DoTT/DoG transcription cannot be explained by differential alterations in transcriptional activity.

Next, we performed hierarchical clustering of genes based on read-through (average of replicates) for each condition (Fig 3B). This identified a large cluster of 1,368 genes (37%) with read-through in all conditions (marked in blue) as well as a number of clusters with differences between conditions. It furthermore highlighted the prevalence of DoTT/DoG transcription with only 102 genes (3%) showing no DoTT/DoG transcription (defined as ≤5% read-through) in any infected/stress sample. Overrepresentation analysis for Gene Ontology (GO) terms using DAVID [20] found an enrichment of genes with extracellular regions (25 genes) and heparin binding (6 genes) among these 102 genes. However, no functional categories were overrepresented for the 1,368 genes with read-through in all conditions.

Interestingly, the only gene experiencing ≥75% read-through already after 2-3h p.i. HSV-1 infection and in all stress conditions was interferon regulatory factor 1 (IRF1) (Fig H in S3 File). IRF1 is an important mediator of both type I and II interferon signaling and studies with IRF1-deficient mice have shown an important role for IRF1 in the immune response against viruses [21–23]. Furthermore, even a relatively small reduction in IRF1 expression, e.g. mediated by cellular miR-23a, is sufficient to measurably augment HSV-1 replication in cell culture [24]. Notably, ribosome profiling data from our previous study revealed a >4-fold drop in IRF1 translation during HSV-1 infection despite an >1.8-fold increase in total RNA at 8h p.i. [10]. This presumably reflects the negative effects of DoTT on IRF1 translation and suggests that HSV-1 exploits DoTT to evade the host immune response.

A striking characteristic of HSV-1-induced DoTT was the associated increase in aberrant splicing [10]. In particular, this comprised novel intragenic and intergenic splicing events as well as splicing associated with nonsense-mediated decay (NMD). Intergenic splicing joins known exons of neighboring genes and confirms transcription of large chimeric transcripts spanning two or more cellular genes. It can be observed as early as 3-4h p.i. in HSV-1 infection. One of the most prominent examples connects SRSF2 and JMJD6. We also observed intergenic splicing in the two stress conditions, but the few examples did not cluster with intergenic splicing events in HSV-1 infection (Fig 3C). Analysis of induced splicing events upstream of gene 3’ ends, however, showed similar characteristics in both HSV-1 infection and salt and heat stress. In all three conditions, induced intragenic splice junctions were enriched for novel splice junctions and junctions found only in processed transcripts (containing no ORF but not classified as long or short non-coding RNAs) or in NMD-associated transcripts (Fig 3D; examples in Fig I in S3 File). Genes with induced intragenic splicing events showed increased read-through in all three conditions (Fig I in S3 File), but read-through was also observed in genes without induced splicing events. Thus, aberrant splicing upstream of gene 3’ ends more likely resulted from, rather than is responsible for DoTT/DoG transcription. One possible explanation for the association of aberrant splicing with DoTT/DoG transcription may be that all serine and arginine rich splicing factor (SRSF) genes included in our analysis (SRSF2, SRSF3, SRSF5, SRSF6, SRSF7, SRSF10, SRSF11) showed DoTT/DoG transcription in at least two, but mostly all three conditions. All of these SRSF genes showed a >2-fold greater drop in translation at 8h p.i. HSV-1 infection in the ribosome profiling data than expected from the changes in their total RNA levels.

### Role of Ca^2+^ release and IP3R in DoTT/DoG transcription

**Fig 4:**
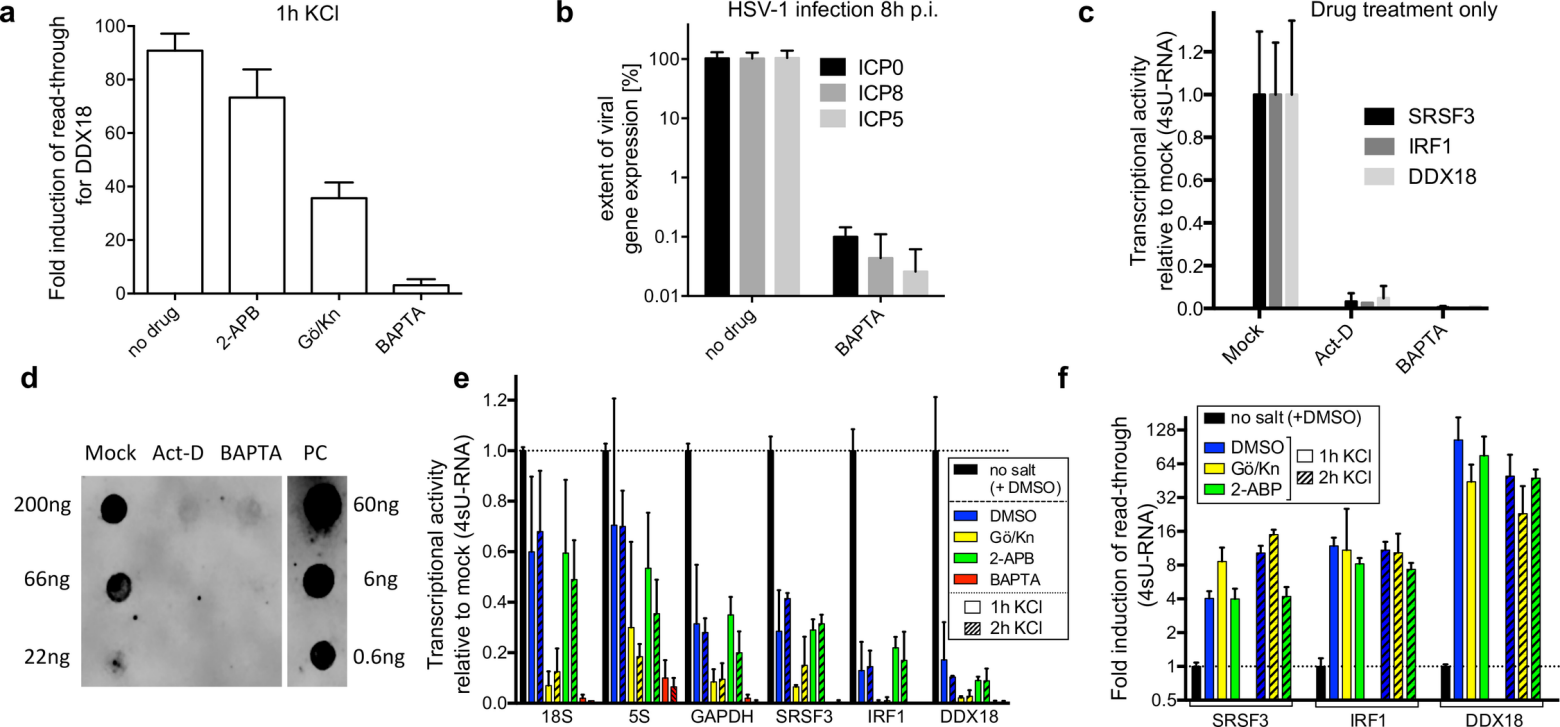
Role of Ca^2+^ release and IP3R in DoTT/DoG transcription. (a) HFF were exposed to 1h salt stress in presence of (i) an inhibitor of IP3R signaling (2-APB), (ii) the membrane permeable Ca^2+^ chelator BAPTA-AM, or (iii) inhibitors of CaMKII and PKC/D (KN93 and Gö6976, respectively, Gö/Kn in Figure). DoTT/DoG transcription was quantified in total cellular RNA by qRT-PCR for DDX18. (b) HFF were infected with wildtype HSV-1 at an MOI of 10 and BAPTA-AM was added at 1h p.i. to the cell culture medium. Viral gene expression of ICP0 (immediate-early), ICP8 (early) and ICP5 (late kinetics) were quantified at 8h p.i. by qRT-PCR. (c) The effect of BAPTA-AM on Pol II activity was determined for three genes in 4sU-RNA obtained following 60min of 500μM 4sU-tagging in presence of either Act-D (positive control) or BAPTA-AM. Cells were pretreated with either of the two drugs or DMSO for 30min prior to 4sU-tagging. For all experiments (a-c), combined data of three biological replicates are shown. (d) Effects of BAPTA-AM on global transcription rates were determined by dot blot analysis of thiol-specifically biotinylated total cellular RNA obtained from cells following 60min of 500µM 4sU exposure in presence of BAPTA-AM, Act-D (global inhibition of transcription) or mock. A biotinylated DNA oligo (1biotin in 40nt) served as positive control (PC). A representative of three independent experiments is shown. (e) The effect of BAPTA-AM, 2-APB, and Gö6976 / KN93 co-treatment on Pol I (18S rRNA), Pol II (GAPDH, SRSF3, IRF1, DDX18) and Pol III (5S rRNA) transcriptional activity were quantified in purified 4sU-RNA obtained following 60min of 4sU-tagging during the first and second hour of salt stress. Equal amounts of input RNA (60μg biotinylated RNA) were used to purify 4sU-RNA and data were normalized to this. (f) In the same experiment, transcriptional activity downstream of SRSF3, IRF1 and DDX18 was quantified by qRT-PCR and compared to transcription rates within the respective gene bodies. Data from two biological replicates are shown. BAPTA-AM treatment did not allow recovering sufficient amounts of RNA for quantification.

Vilborg et al. reported that salt stress-induced DoG transcription in SK-N-BE(2)C cells depends on IP3R activation, Ca^2+^ release from intracellular stores and downstream kinases [11]. HSV-1 entry into cells is dependent on the activation of Ca^2+^ signaling pathways and triggers Ca^2+^ release from intracellular stores [25, 26]. In addition, HSV-1 infection results in an increasing loss of stable, resting Ca^2+^ at late times of infection indicating a bimodal role of Ca^2+^ signaling in HSV-1 infection [27]. Before assessing the effect of Ca^2+^ signaling inhibitors on DoTT in HSV-1 infection of HFF, we first aimed to reproduce the results by Vilborg et al. in salt stress. HFF were exposed to 80mM KCl for 1h in presence of (i) an inhibitor of IP3R signaling (2-APB), (ii) the membrane permeable Ca^2+^ chelator BAPTA-AM, or (iii) inhibitors of the downstream kinases Ca^2+^/calmodulin-dependent protein kinase II (CaMKII) and protein kinase C/protein kinase D (PKC/D) (KN93 and Gö6976, respectively). DoG transcription was first quantified by qRT-PCR on total RNA for DDX18, which shows strong read-through in HSV-1 infection as well as salt and heat stress. Consistent with the previous report, BAPTA-AM prevented DoG transcription while the other inhibitors resulted only in a moderate (25–65%) reduction (Fig 4A). We thus aimed to assess the effect of BAPTA-AM on DoTT in HSV-1 infection. To avoid the described detrimental effects of BAPTA-AM on virus entry and the onset of productive infection [25, 26], we only added BAPTA-AM to the cell culture media of HFF at 1h p.i. (MOI = 10) when viral gene expression is already well initiated. To first determine its effect on viral gene expression, we quantified immediate-early (ICP0), early (ICP8) and true late (ICP5) gene expression at 8h p.i. by qRT-PCR. Strikingly, BAPTA-AM treatment was highly detrimental to viral gene expression of all three kinetic classes resulting in a >1,000-fold drop in viral mRNA levels (Fig 4B).

Considering this strong reduction in viral gene expression, we hypothesized that depletion of intracellular Ca^2+^ by BAPTA-AM in HFF might globally impair Pol II activity rather than specifically interfere with DoTT/DoG transcription. We thus analyzed the effect of 1h of BAPTA-AM treatment of uninfected cells on transcriptional activity of three cellular genes (SRSF3, IRF1 and DDX18). For this purpose, we labeled newly transcribed RNA by adding 500μM 4sU to the cell culture medium for 1h. Following isolation and purification of the 4sU-labeled newly transcribed RNA (4sU-RNA) from a fixed amount of biotinylated total RNA per condition (60μg), transcriptional activity of these genes was quantified using qRT-PCR on 4sU-RNA. BAPTA-AM indeed induced a drop in transcriptional activity that was at least as strong as observed upon inhibition of Pol II using actinomycin D (Act-D; Fig 4C). In addition, global 4sU incorporation rates into total cellular RNA were substantially reduced upon BAPTA-AM treatment (Fig 4D). This indicated that BAPTA-AM might not only interfere with Pol II but also with rRNA synthesis (Pol I and III transcription), which contributes about 50–60% of 4sU-RNA in HFF as estimated from our RNA-seq data [10]. We thus quantified transcription rates from 4sU-RNA for a Pol I transcript (18S rRNA), a Pol III transcript (5S rRNA) in addition to four genes transcribed by Pol II (GAPDH, SRSF3, IRF1 and DDX18) upon exposure of HFF to 80mM KCl for 1 and 2h and BAPTA-AM (Fig 4E). In addition, we tested whether the combined exposure of cells to Gö6976 and KN93, which also diminished salt stress-induced DoG transcription in total RNA, also globally affected transcriptional activity. While salt stress alone already resulted in a drop in transcription rates for Pol I (≈1.5-fold), II (3- to 5-fold) and III (≈1.4-fold) transcripts, BAPTA-AM impaired transcriptional activity of all three polymerases. This suggests that global inhibition of cellular RNA polymerases by BAPTA-AM rather than a specific effect on transcription termination is responsible for the loss of salt stress-induced DoG transcripts. As BAPTA and its derivatives share a high selectivity for Ca^2+^ over Mg^2+^ (>10^5^ stronger binding), the observed effects did not result from the co-depletion of intracellular Mg^2+^ [28]. Interestingly, combined Gö6976/KN93 treatment also globally impaired Pol I, II and III transcription, albeit to a lesser degree (2- to 10-fold), thereby explaining the slight reduction in DoG levels in total RNA (Fig 4A). In contrast, 2-ABP treatment, which had shown no effect on DoG transcription when analyzing total cellular RNA, did not impair polymerase activity. Finally, we quantified read-through transcription for the three DoG genes SRSF3, IRF1 and DDX18 in 4sU-RNA (Fig 4F). Neither KN93/Gö6976 nor 2-ABP treatment had any effect on the induction of the respective DoG transcripts. Unfortunately, BAPTA-AM treatment did not allow to reliably measure read-through transcription due to the impaired transcription (very low copy numbers or even negative PCR results). We conclude that the reduced levels of DoG transcripts upon inhibition of Ca^2+^ signaling do not result from direct effects on DoG transcription but from global inhibitory effects on cellular transcription in general. To our knowledge, this strong inhibitory effect of BAPTA-AM treatment on RNA polymerase activity has not been appreciated so far and should be considered when interpreting results obtained using BAPTA-AM to inhibit calcium signaling.

### Subcellular RNA localization of read-through transcripts in HSV-1 infection

Vilborg et al. initially reported that DoG transcripts (DoGs) were strongly enriched at the chromatin [11] and that one of the more abundant DoGs, *doSERBP1* (*d*ownstream *o*f SERBP1), remained at the site of synthesis. However, they subsequently also observed DoGs in the nucleoplasma of cells when searching for them by confocal microscopy with increased sensitivity [13]. To assess the fate of the transcripts arising from DoTT in HSV-1 infection, we separated cell lysates (uninfected cells and 8h p.i.) into cytoplasmic, nucleoplasmic and chromatin-associated fractions [29, 30] and analyzed all three fractions as well as total cellular RNA by RNA-seq (2 replicates). The efficient separation of the cytoplasmic from the nuclear RNA fraction was confirmed by the enrichment of well-described nuclear lincRNAs (MALAT1, NEAT1, MEG3; Fig J in S3 File) in nucleoplasmic and chromatin-associated RNA as well as cytoplasmic enrichment of reported cytoplasmic lincRNAs (LINC00657, VTRNA2-1; Fig J in S3 File). In addition, overrepresentation of intronic reads in chromatin-associated RNA compared to nucleoplasmic RNA (>5-fold higher) demonstrated the efficient separation of these two RNA fractions (Fig J in S3 File)

**Fig 5:**
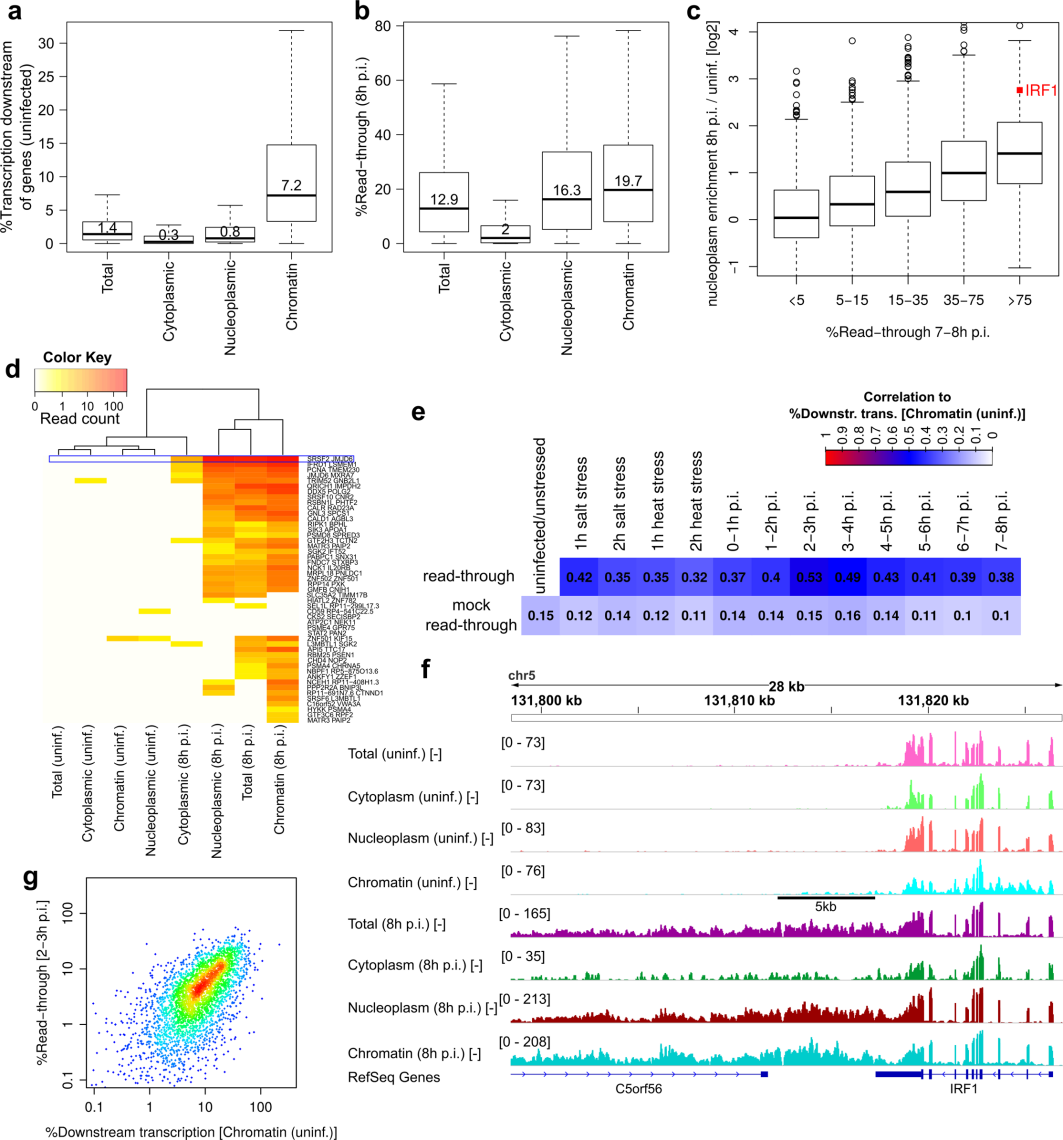
Subcellular localization of read-through transcripts in HSV-1 infection. (a) Boxplots indicating the distribution of the percentage of transcription downstream of genes identified before infection in total, cytoplasmic, nucleoplasmic and chromatin-associated RNA. Numbers in boxes indicate median values. (b) Boxplots indicating the distribution of read-through at 8h p.i. HSV-1 infection in all RNA fractions. Numbers in boxes indicate median values. (c) Boxplots indicating the distribution of log2 ratios of nucleoplasm enrichment (= gene FPKM in nucleoplasmic RNA/ gene FPKM in cytoplasmic RNA) at 8h p.i. compared to uninfected cells. Ratios are shown separately for groups of genes with different amounts of read-through in 7-8h p.i. 4sU-RNA. The value for IRF1 is highlighted in red. (d) Heatmap of read counts (sum of 2 replicates) in total, cytoplasmic, nucleoplasmic and chromatin-associated RNA for the intergenic splicing events shown in Fig 3C. Results for the intergenic splicing junction connecting SRSF2 and JMJD6 are highlighted by a blue box. (e) Spearman correlation between read-through (calculated from 4sU-seq data) in all conditions and the percentage of transcription downstream of genes identified in chromatin-associated RNA of uninfected/untreated cells. Correlation to mock read-through values is shown below. Mock read-through values were calculated as described in methods and correlations were averaged for each condition. (f) Mapped sequencing reads (negative strand) for total (light/dark pink), cytoplasmic (light/dark green), nucleoplasmic (light/dark red) and chromatin-associated RNA (light/dark cyan) in uninfected cells (light colors) and at 8h p.i. (dark colors) for the IRF1 gene. Read coverage ranges and RefSeq gene annotation are indicated as in Fig 1C. (g) Scatterplot of read-through at 2-3h p.i. against the percentage of transcription downstream of genes identified in chromatin-associated RNA of uninfected/untreated cells. Colors indicate density of points (red = highest density, blue = lowest density).

In uninfected cells, only chromatin-associated RNA showed notable levels of downstream transcription (median 7.2%; Fig 5A), consistent with the standard model of transcription termination in eukaryotic cells [1]. At 8h p.i., substantial read-through was observed in all fractions except for cytoplasmic RNA (Fig 5B, Table B in S1 File), indicating that read-through transcripts are not efficiently exported to the cytoplasm. When we grouped genes according to their extent of read-through in 7-8h p.i. 4sU-RNA, we observed a strong increase during infection in the enrichment of the respective mRNAs (counting only the exonic regions upstream of gene 3’ ends) in both nucleoplasmic (Fig 5C) and chromatin-associated RNA (Fig J in S3 File) depending on the extent of read-through. While no change in nuclear enrichment was observed for genes without read-through, genes with >75% read-through were on average >2.5-fold more enriched at 8h p.i. than in uninfected cells. In particular, IRF1 was >6 and 4-fold more enriched in nucleoplasmic and chromatin-associated RNA, respectively, at 8h p.i. than in uninfected cells. Further evidence for an inefficient export of read-through transcripts is provided by intergenic splicing events, which are mostly absent in cytoplasmic RNA at 8h p.i. despite their considerable abundance in the other subcellular RNA fractions (Fig 5D). This also explains our previous observation based on ribosome profiling that RNA chimeras and consequently genes induced by read-in transcription arising from DoTT are not, or only poorly translated [10]. We conclude that DoTT leads to nuclear retention of the respective read-through transcripts and thereby notably contributes to HSV-1 induced host shut-off.

The similar overall level and high gene-specific correlation ($R_{s}\;=\;0.8$) of read-through in nucleoplasmic and chromatin-associated RNA indicates that transcripts resulting from HSV-1-induced DoTT are generally released from the chromatin, i.e. the site of synthesis, into the nucleoplasm (see e.g. Fig 5F). Nevertheless, we identified 18 genes (Table C in S1 File) for which these transcripts appeared to remain at the chromatin (≤5% read-through in nucleoplasmic and cytoplasmic RNA, but ≥25% in chromatin-associated RNA; examples in Fig K in S3 File).

Interestingly, there was a modest correlation ($R_{s}\;=\;0.32-0.53$) between the percentage of downstream transcription observed in chromatin-associated RNA of uninfected/unstressed cells and read-through upon stress or HSV-1 infection (Fig 5E). This suggests that genes with a relatively high extent of downstream transcription in uninfected/unstressed cells might be predisposed for DoTT/DoG transcription. To exclude that this was an artifact of read-through being calculated from downstream transcription, we calculated ‘mock’ read-through values from the two biological replicates for the same time-point (see methods). For mock read-through, the correlation was much lower at only ~0.13. This suggests a link between downstream transcription detectable in chromatin-associated RNA in uninfected/untreated cells and read-through in stress/infection. A possible explanation might be that the respective poly(A) sites are weaker and thus more prone to further disruption by HSV-1 or stress-related mechanisms. Fig 5F illustrates this for IRF1, for which downstream transcription in chromatin-associated RNA of uninfected cells was 14% and covered ~5kb. Interestingly, the correlation between downstream transcription in chromatin-associated RNA in uninfected cells and read-through during infection was highest at early time-points, i.e. at 1h for salt/heat stress and 2-3h p.i. for HSV-1 infection (Fig 5E and 5G). At late stages of HSV-1 infection, even cellular genes with very little downstream transcription in chromatin-associated RNA from uninfected cells showed read-through transcription (Fig J in S3 File).

### DoTT increases chromatin accessibility downstream of cellular genes

Based on publicly available DNase hypersensitive and ATAC-seq data for unstressed murine fibroblasts, Vilborg et al. recently reported that, even prior to stress, pan-DoG genes are already characterized by a chromatin signature indicative of an open chromatin state. However, due to the lack of respective data following salt or heat stress, they could not assess the consequences of read-through on cellular chromatin. We thus performed ATAC-seq in HFF at 0, 1, 2, 4, 6 and 8h of HSV-1 infection and 1 and 2h of salt and heat stress (n = 2). For all ATAC-seq samples, open chromatin regions (OCRs) were enriched around promoters, thereby confirming the high quality of the data (Fig L in S3 File). Both length and score of OCRs at gene promoters correlated with gene expression in uninfected cells ($R_{s}\;=\;0.42$ and 0.4, respectively; Fig L in S3 File). In contrast to the findings by Vilborg et al., we did not observe a positive correlation between DoTT/DoG transcription and the presence of OCRs in the 5kb downstream of genes in unstressed/uninfected cells (Fig M in S3 File). However, we noted a weak positive correlation ($R_{s}\leq 0.25$) between the presence of downstream OCRs (dOCRs) and the expression level of the corresponding genes (Fig M in S3 File). Notably, the highly expressed genes GAPDH and ACTB, which were not affected by DoTT/DoG transcription (Fig 1D; Fig A in S3 File), were characterized by open chromatin downstream of their 3’ends already in uninfected cells (Fig M in S3 File). In summary, our data argues against genes being predisposed for DoTT/DoG transcription by open chromatin downstream of their 3’ ends.

**Fig 6:**
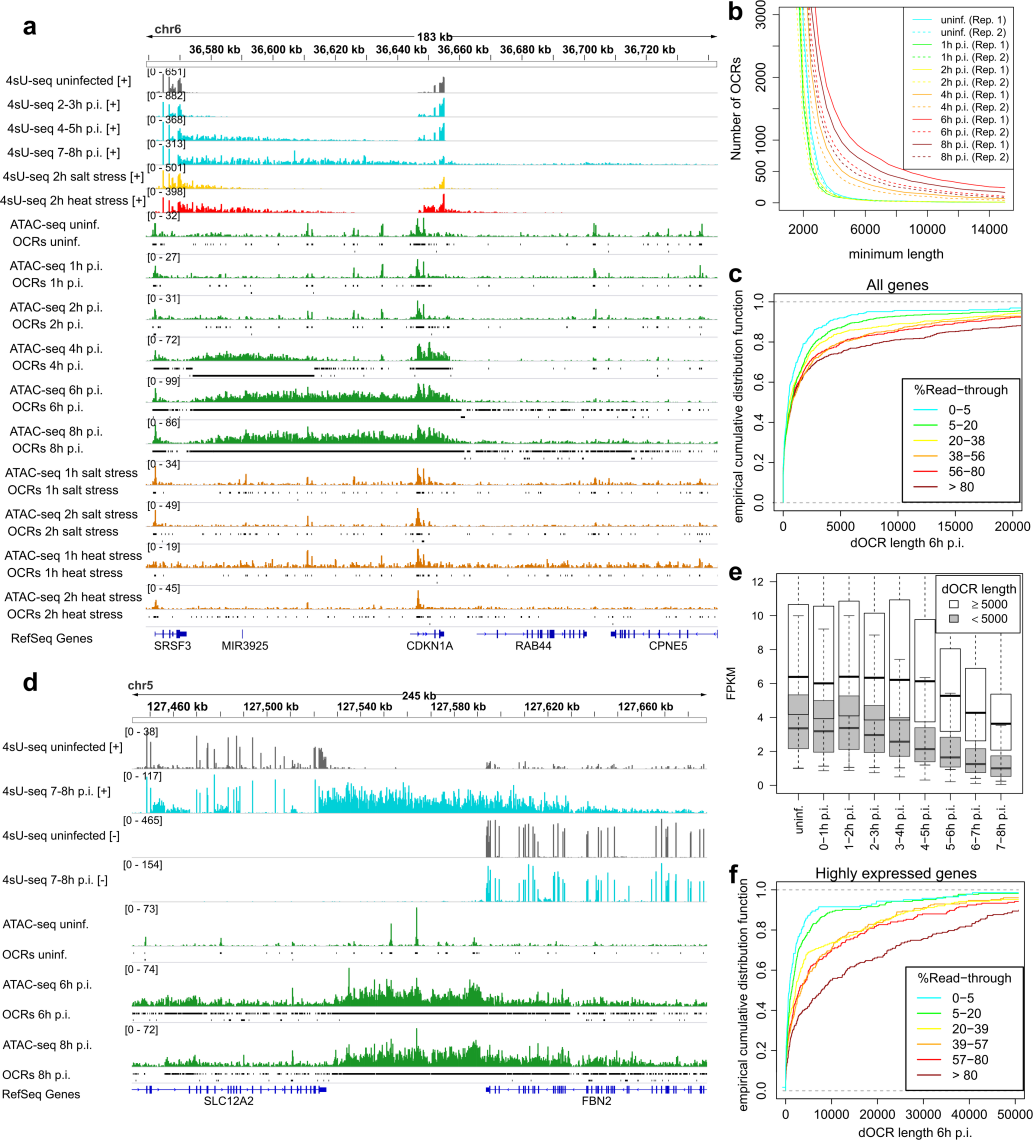
Extensive increase of downstream open chromatin during HSV-1 infection. (a) 4sU-seq read coverage for SRSF3 (strand-specific, grey = uninfected/unstressed, cyan = selected time-points of HSV-1 infection, yellow = 2h salt stress, red = 2h heat stress) as well as ATAC-seq data (no strand specificity, green = HSV-1 infection, brown = salt and heat stress) and identified open chromatin regions (OCRs, black lines). The OCRs shown here were derived from replicate 1. Read coverage ranges and RefSeq gene annotation are indicated as described in Fig 1C. (b) Numbers of identified OCRs (y-axis) with a certain minimum length (x-axis) for all ATAC-seq samples. Results for replicates are shown separately (solid lines = replicate 1, dashed lines = replicate 2). (c) Empirical cumulative distribution functions indicating the fraction of genes (y-axis) with at most a certain dOCR length (average between two replicates) at 6h p.i. (x-axis). Genes were grouped according to read-through in 7-8h p.i. as described in methods. (d) 4sU-seq and ATAC-seq read coverage and identified OCRs in HSV-1 infection for the FBN2 and SLC12A2 genes. 4sU-seq reads are shown separately for positive [+] and negative [–] strand. ATAC-seq reads are not strand-specific. Read coverage ranges and RefSeq gene annotations are indicated as in Fig 1C. (e) Boxplots indicating the distribution of gene expression (FPKM) values for genes with >80% read-through at 7-8h p.i. and dOCR length of either ≥5kb (white) or <5kb (gray.) (f) Empirical cumulative distribution functions indicating the fraction of genes (y-axis) with at most a certain downstream dOCR length at 6h p.i. (x-axis) for highly-expressed genes (FPKM at 7-8h p.i. ≥2). Genes were grouped according to read-through in 7-8h p.i. as described in methods.

We next analyzed the impact of HSV-1-induced DoTT on chromatin accessibility. To our surprise, we observed a substantial increase in open chromatin downstream of individual genes with HSV-1-induced DoTT (Fig 6A, Fig N in S3 File). Here, downstream regions were often covered by OCRs for tens-of-thousands of nucleotides, similar to the pattern of read-through transcription in these downstream regions. This already became detectable at 4h p.i and resulted in a substantial increase in the number of long OCRs (Fig 6B), which were specifically enriched downstream of genes (Fig O in S3 File). Thus, these do not result from global effects of HSV-1 infection on cell viability (e.g. due to enhanced chromatin accessibility in a subpopulation of dying cells). To quantify the total extent of open chromatin downstream of individual genes, we assigned dOCRs to genes if they were either close to the gene 3’ end or another dOCR that had already been assigned to the respective gene (see methods) and then calculated the total genomic length covered by dOCRs (= dOCR length). This revealed a specific increase of dOCR length throughout infection for genes with high read-through (Fig 6C). For 174 of the 681 genes (26%) with >80% read-through at 7-8h p.i., dOCR length exceeded 5kb at 6h p.i., while only 26 of 326 (8%) genes with ≤5% read-through at 7-8h p.i. had a dOCR length ≥5kb at 6h p.i. (Fisher’s exact test $p\;=\;6.71\times 10^{-12}$). For 11 of these 26 genes (42%), this was likely due to a close-by downstream gene with DoTT on the opposite strand (see Fig 6D for FBN2, >60kb dOCR matches the read-through of the SLC12A2 gene on the opposite strand). These 11 genes showed no DoTT despite long dOCRs (originating from DoTT for genes with convergent transcription on the opposite strand) and strong expression at 7-8h p.i. (10 with FPKM >1, 6 with FPKM >3). This indicates that the increase in downstream open chromatin during HSV-1 infection is not responsible for DoTT but rather that the formation of dOCRs requires DoTT. Furthermore, induction of long dOCRs for genes with read-through was dependent on the transcription rates of the respective genes. Genes with >80% read-through and long dOCRs were much higher expressed at 7-8h p.i. than read-through genes without long dOCRs (Fig 6E). Accordingly, when dOCR length was compared to read-through for the 1,273 most highly expressed genes (FPKM ≥2) at 7-8h p.i., the difference in dOCR length between genes with different read-through levels was much more pronounced (Fig 6F). Finally, strong increases in OCRs within gene bodies or promoter regions were only observed for genes with read-in transcription but not upstream of the poly(A) read-through. This explains the smaller, but nevertheless notable global increase in long OCRs in gene bodies (Fig O in S3 File).

Given the striking increase in dOCR length for well-expressed genes affected by HSV-1 induced DoTT, we also expected to see an increase in chromatin accessibility for salt and heat stress. However, there was no general increase in the number of long OCRs during salt or heat stress and no increase in dOCR length for individual genes in contrast to HSV-1 infection (Fig P in S3 File). Accordingly, dOCR length did not increase for genes with high levels of stress-induced DoG transcription (Fig P in S3 File), not even for highly expressed genes (Fig 6A, Fig N in S3 File). Since read-through at 2h salt and heat stress was comparable to 4-5h p.i. HSV-1 infection and extensive dOCRs were clearly detectable at 4h p.i., stress-induced DoG transcription does not appear to lead to open chromatin downstream of genes. Thus, only HSV-1 induced DoTT, but not DoG transcription in salt or heat stress, results in this striking increase in the accessibility of genomic regions downstream of affected genes.

### Histone modifications and DoTT/DoG transcription

In addition to enhanced chromatin accessibility downstream of pan-stress DoGs, Vilborg et al. also found an enrichment of several histone marks typically found at actively transcribed genes (H3K36me3, H3K79me2) and at enhancers (H3K4me1, H3K27ac) based on ENCODE data from unstressed murine NIH-3T3 fibroblasts [13]. Considering the discrepancy of our findings regarding open chromatin to their findings, we also analyzed ChIP-seq data from ENCODE for histone marks in uninfected/unstressed HFF. Significant positive correlations (FDR adjusted p-value <0.01) between read-through in stress conditions and the presence of histone marks in the 5kb downstream of genes were only observed for the elongation marker H3K36me3 and DoG transcription in heat stress (Fig M in S3 File). However, weak positive but not significant correlations were also observed in salt stress for H3K36me3. The same was true in both stresses for two markers of accessible regulatory chromatin, H3K27ac and H3K4me1. Interestingly, for H3K36me3, positive correlations were also observed to read-through already detectable in the first two hours of HSV-1 infection. However, at later times of infection, this shifted to highly significant negative correlations between read-through and the presence of H3K27ac, H3K27me3, H3K4me1 and H3K4me3 marks in the ENCODE data for uninfected cells. While this highlights important differences between DoTT and DoG transcription, the biological significance of the presence of certain histone marks in cells prior to stress or infection remains unclear. ChIP-seq data from time-course experiments of both HSV-1 infection and stress will be required to resolve these conflicting observations.

### HSV-1-activated antisense transcription, DoTT and dOCRs

In collaboration with Wyler et al., we recently reported on the activation of antisense transcription in the human genome during lytic HSV-1 infection [14]. To assess whether this antisense transcription was also associated with the formation of OCRs, we investigated the 11 antisense transcripts that had been extensively validated by RT-qPCR and Nanostring nCounter assays (Fig Q in S3 File). Interestingly, induction of antisense transcripts was clearly accompanied by an induction of corresponding long OCRs in three of these cases (BBCas, EFNB1as, ING1as). These represented 3 of the 4 (together with C1orf159as) most highly expressed antisense transcripts at 7-8h p.i., consistent with a role of transcription in the formation of long OCRs. For another four cases, an effect on open chromatin was visible but less clear (NFKB2as, IFFO2as, FOXO3as, C1orf159as). Moreover, similar to transcripts of DoTT-affected genes, the length of the 11 antisense transcripts gradually increased quite substantially during HSV-1 infection, indicating that they are also affected by DoTT. To exclude that long OCRs during HSV-1 infection are an artifact of or are directly related to the induced antisense transcription, we determined the fraction of long OCRs (≥5kb; <80 long OCRs per replicate in uninfected cells, >500 per replicate at 6 and 8 p.i.) that overlapped (≥25% of OCR in antisense transcript) any of the 3,098 antisense transcripts identified by Wyler et al. (Fig R in S3 File). In uninfected cells, ≥40% of the few long OCRs overlapped with an antisense transcript. With increasing duration of infection, this fraction decreased and only ~13% of long OCRs overlapped an antisense transcript at 8h p.i., but often also a region of read-through transcription on the opposite strand. This supports a model in which HSV-1-induced dOCRs originate from read-through transcription while antisense transcripts also experience DoTT and consequently show the associated long OCRs if transcribed at a sufficient rate.

## Discussion

HSV-1 infection, cellular stress responses and cancer result in extensive transcriptional activity downstream of a subset of cellular genes [10–12], but the relationships between the underlying molecular mechanisms remained unclear. By directly comparing HSV-1-induced DoTT with DoG transcription in salt and heat stress in the same experimental setting, we show significant overlaps between the genes affected by DoTT/DoG transcription but also clear context- and condition-specific differences. Importantly, differences were not only observed between DoTT and DoG transcription but also for DoG transcription between the two different stresses. Notably, the gene-specific correlation of read-through between salt stress and heat stress essentially equaled the correlation between salt stress and HSV-1 infection at 4-5h p.i. Multiple cis- and trans-regulatory factors are known to determine both splicing and poly(A) site usage [31] and even promoter elements have been shown to shape RNA processing by influencing Pol II processivity [32]. Thus, variability in DoG transcription upon different stressors and HSV-1-induced DoTT may originate from differences in downstream responses, interactions with other signaling pathways activated upon the different stresses or infection or even activation of alternative pathways with similar molecular consequences on the transcription termination machinery. In any case, the striking similarities between DoTT and DoG transcription indicate that related mechanisms are at play during HSV-1 infection. While the extent of DoTT further increased at late times of infection, salt stress-induced DoG transcription already declined by 2h, presumably due to detrimental effects of prolonged exposure to enhanced extracellular K^+^ concentrations on the exposed cells. In this respect, the expression of viral proteins counteracting the consequences of detrimental stress responses such as translational arrest and apoptosis may enable the much more efficient disruption of transcription termination by HSV-1.

The results presented here and in our previous manuscript [10] could have been a result of transcriptional noise that becomes evident in the context of transcription inhibition or extensive degradation of actively transcribed mRNAs by the virion-associated host shut-off protein (*vhs*) [33]. Alternatively, it might result from *de novo* pervasive transcription initiation downstream of the respective genes. However, we disfavor these models. First, it is important to note that we analyzed newly transcribed rather than total RNA. Therefore, transcriptional activity downstream or upstream of genes is always directly compared to the transcriptional activity of the corresponding gene occurring during the same timeframe of infection. The global loss in Pol II activity should equally affect genomic regions within, downstream and upstream of genes. Furthermore, strong transcriptional down-regulation of hundreds of genes has been analyzed in a broad range of different conditions using 4sU-seq [34–36], none of which showed any increase in transcriptional activity downstream of genes. In addition, infection with a *vhs*-null mutant, which does not trigger a notable decline in transcriptional activity until at least 12h of infection [17], still resulted in a very similar extent of read-through transcription as wild-type HSV-1 infection [10].

The data obtained in this study provide further strong evidence that downstream transcriptional activity arises from DoTT. First, the high correlations between the extent of read-through in HSV-1 infection, salt and heat stress indicate that all three conditions involve a common mechanism, namely poly(A) read-through. Second, RNA-seq analysis of subcellular RNA fractions revealed a striking dependence of nuclear retention of exonic regions during infection on the extent of read-through observed for the respective genes. The most likely scenario is that DoTT and extensive poly(A) read-through transcription result in large aberrant transcripts that cannot be efficiently exported to the cytoplasm. Third, the induction of extensive dOCRs for genes experiencing DoTT, which depends on the transcription level of these genes, provides strong evidence for an increase in absolute transcriptional activity downstream of these genes during infection. Additional evidence against pervasive *de novo* transcription initiation downstream of genes is provided both by the intergenic splicing events between neighboring genes induced in HSV-1 infection and the strong strand-specificity of the downstream transcriptional activity. D*e novo* transcription initiation would not be limited to the strand of the upstream gene but would be expected to occur on either strand. The strong strand-specificity also excludes that downstream transcriptional activity is an artifact of the reported activation of antisense transcription during infection [14]. Moreover, DoTT and read-through transcription is clearly much more prominent than this antisense transcription. We now even provide evidence that antisense transcripts are also affected by DoTT and show DoTT-associated dOCRs.

Vilborg et al. reported that DoG transcription was associated with an open chromatin state downstream of genes prior to stress [11]. This observation was not confirmed in our ATAC-seq data from primary human fibroblasts. While we currently cannot fully explain the discrepancy between these findings, we hypothesize that the enrichment of accessibility marks observed by Vilborg et al. may result from a restriction to pan-DoG genes detectable in nuclear RNA. As this also includes RNA transcribed before stress, relative levels of DoG transcripts are lower than in newly transcribed RNA. Thus, their analysis may be biased towards more highly transcribed DoG transcripts, which are more readily detectable. When we analyzed histone mark ChIP-seq data from ENCODE for uninfected HFF, we could only reproduce the positive correlation reported by Vilborg et al. [13] between the presence of the transcription elongation mark H3K36me3 (but not H3K4me1 and H3K27ac) and read-through in heat stress and to a lesser degree in salt stress. Interestingly, this was also observed during the first two hours of HSV-1 infection, which nicely fits to our observation that genes with active downstream transcription in chromatin-associated RNA in uninfected cells are more prone to read-through. In addition, it indicates that read-through occurring very early in HSV-1 infection may reflect a cellular stress response to infection and thus essentially DoG transcription. Later in infection, however, the picture completely shifts to negative correlations between read-through and repressive (H3K27me3) and general (H3K4me3) promoter marks as well as accessible regulatory chromatin (H3K27ac and H3K4me1). While the correlation with both repressive promoter marks and activating histone marks late in HSV-1 infection is difficult to interpret and seems contradictory, it hints that at this point other mechanisms than a general stress response may come into play. It is important to note, however, that the respective ChIP-seq data were only obtained from uninfected/unstressed cells and thus do not reflect the changes in histone marks upon infection/stress.

The most striking finding of our study is the extensive increase in genome accessibility downstream of well-expressed genes affected by DoTT during HSV-1 infection, which essentially matched the transcriptional read-through observed at the respective time of infection. Of note, the peak heights of extensive dOCRs were often similar to levels observed in gene promoters of the respective genes where histones are displaced by transcription factors binding to promoter elements. However, in DoTT-associated dOCRs, this was not restricted to a few hundred base pairs but extended for tens-of-thousands of nucleotides. Our data indicate that dOCRs are not the cause but rather the consequence of DoTT and their formation additionally requires high levels of transcriptional activity in the respective downstream genomic regions. Considering the high correlation between DoTT and DoG transcription, we were surprised not to observe any evidence of dOCRs for DoG transcription in salt or heat stress. As DoTT-associated dOCRs were already well detectable by 4h of infection when the overall extent of DoTT and DoG transcription was very similar, the lack of dOCRs in salt and heat stress is not merely due to quantitative differences between the three conditions.

Progression of transcribing Pol II across a gene is accompanied by the displacement of nucleosomes, followed by their rapid co-transcriptional repositioning immediately behind Pol II (reviewed in [37]). We hypothesize that dOCRs result from impaired histone repositioning in the wake of Pol II. The lack of dOCRs in salt and heat stress indicates that dOCRs do not merely arise when Pol II starts transcribing far into previously untranscribed regions of the genome. Furthermore, gene bodies upstream of poly(A) sites affected by DoTT showed no general induction of OCRs, suggesting that there is no general inhibition of histone repositioning during HSV-1 infection. However, induced OCRs were also observed in gene bodies following read-in transcription, which argues against a role of distinct histone modifications in intergenic regions. Interestingly, HSV-1 infection was found to mobilize histones including linker and core histones (H1, H2B, H3.1 and H4) as well as histone variants (H3.3) [38, 39]. This resulted in increases in the pools of “free” histones despite an inhibition of histone synthesis during infection [40, 41]. Therefore, it is unlikely that the induction of dOCRs results from a deprivation of free histones. On the contrary, the reported histone mobilization may at least partly result from impaired histone repositioning downstream of genes and thus release of histones from the respective regions into the nucleoplasm following read-through transcription. A critical role in nucleosome reassembly is played by the histone chaperons Spt6 and the FACT (FAcilitates Chromatin Transcription) complex (reviewed in [42]). Interestingly, recruitment of Spt6 to active cellular genes includes direct interactions with the C-terminal domain (CTD) of Pol II [43, 44]. Here, specific post-translational modifications of the CTD, which depend on the position of the transcribing Pol II within a gene, govern the functional state and properties of Pol II and its interactions with other factors [45]. Recently, the HSV-1 ICP22 protein was found to relocate both Spt6 and FACT to viral replication compartments. This may limit their availability to Pol II when transcribing cellular genes in HSV-1 infection [46], but does not explain the selective failure in nucleosome reassembly only downstream of genes with read-through. Follow-up studies on recruitment and disengagement of Spt6 and FACT from Pol II upon infection with wild-type HSV-1 and mutant viruses as well as the concurrent analysis of post-translational modifications of the Pol II CTD will provide important insights into the functional regulation of transcription by Pol II and its termination downstream of genes.

In summary, our findings provide a much more detailed picture of the molecular processes involved in DoTT/DoG transcription and point the direction for further studies to elucidate the underlying molecular mechanisms.

## Materials and methods

### Cell culture and infections

Human fetal foreskin fibroblasts (HFF) were purchased from ECACC and cultured in DMEM with 10% FBS Mycoplex and 1% penicillin/streptomycin. HFF were utilized from passage 11 to 17 for all high-throughput experiments. This study was performed using wild-type HSV-1 strain 17. Virus stocks were produced in baby hamster kidney (BHK) cells (obtained from ATCC) as described [10]. HFF were infected with HSV-1 24h after the last split for 15 min at 37°C using a multiplicity of infection (MOI) of 10. Subsequently, the inoculum was removed and fresh media was applied to the cells.

### Salt and heat stress

Salt stress was initiated by adding 80mM KCl to the tissue culture medium. Heat stress was started by replacing the cell culture medium with pre-warmed 44°C medium and culturing the cells for 1 or 2h at 44°C. Newly transcribed RNA was labeled for 1h using 500μM 4-thiouridine (Carbosynth). Total RNA was isolated using Trizol and newly transcribed RNA was purified as described [10]. The IP3R inhibitor 2-APB (100μM, Sigma-Aldrich), the PKC/PKD inhibitor Gö6976 (10μM; Tocris), the CaMKII inhibitor KN-93 (10μM; Tocris) and the calcium chelator BAPTA-AM (50μM; Cayman Chemical) were applied as described [11]. Actinomycin D (2μg/ml, Sigma-Aldrich) was applied at a final concentration of 2μM to inhibit Pol II. Reverse transcription was performed using All-in-One cDNA Synthesis Supermix (Biotool) including a mix of hexanucleotide random primers and poly-dT primers. qRT-PCR was performed using the SYBR Green 2x Mastermix (Biotool) (qRT-PCR primer sequences in Table D in S1 File). Relative quantitation was performed using the ΔΔC_T_ approach. Dot blot analysis was performed as described previously [47] with a few minor changes regarding the detection of 4sU-incorporation into total cellular RNA. Briefly, metabolic labeling of newly transcribed RNA was initiated by adding 500μM 4sU to the cell culture medium together with either 50μM BAPTA-AM, 2μg/ml Actinomycin D or mock (DMSO). Total RNA was isolated using Trizol and thiol-specifically biotinylated using Biotin-HPDP. Following removal of the unincorporated Biotin-HPDP by Chloroform extraction and recovery of the biotinylated RNA by isopropanol/ethanol precipitation, 200ng down to 22ng of biotinylated RNA or 60ng to 0.6 ng of a biotinylated oligo (50bp) were spotted on a positively charged Zeta membrane (Biorad) in alkaline buffer. The membrane was subsequently probed with a Streptavidin-DyLight-680 conjugate and visualized using a LI-COR imaging system.

### Preparation of subcellular RNA fractions

Subcellular RNA fractions (cytoplasmic, nucleoplasmic and chromatin-associated RNA) were prepared combining two previously published protocols [29, 30]. For the detailed protocol see S2 File. The efficiency of the fractionations was controlled by qRT-PCRs for intron-exon junctions for ACTG1 (chromatin-associated vs other three fractions) and western blots for histone H3 (nuclear vs cytoplasmic fraction). Fractionation efficiencies were furthermore confirmed on the RNA-seq data by comparing expression values of known nuclear and cytoplasmic RNAs as well as intron contributions (Fig J in S3 File).

### Library preparation and sequencing

Sequencing libraries were prepared using the TruSeq Stranded Total RNA kit (Illumina). While rRNA depletion was performed for total RNA and all subcellular RNA fractions using Ribo-zero, no rRNA depletion was performed for the 4sU-RNA samples. Sequencing of 75bp paired-end reads was performed on a NextSeq 500 (Illumina) at the Cambridge Genomic Services and the Core Unit Systemmedizin (Würzburg).

### ATAC-seq

HFF were infected for 8h with wild-type HSV-1 at an MOI of 10 or exposed to 1h or 2h of 80mM KCl or 44°C as described above. ATAC-seq was performed according to the original protocol starting with 1x10^5^ cells per condition [15]. ATAC-seq libraries were quantified by Agilent Bioanalyser and sequenced by NextSeq 500 at the Cambridge Genomic Services (75bp paired-end reads).

### Processing of 4sU-seq data

Sequencing adapters were trimmed from sequencing reads using cutadapt [48]. Trimmed sequencing reads were mapped against (i) the human genome (GRCh37/hg19), (ii) human rRNA sequences and (iii) the HSV-1 genome (HSV-1 strain 17, GenBank accession code: JN555585, only for HSV-1 infection data) using ContextMap v2.7.9 [49] (using BWA as short read aligner [50] and allowing a maximum indel size of 3 and at most 5 mismatches). For the two repeat regions in the HSV-1 genome, only one copy each was retained, excluding nucleotides 1–9,213 and 145,590–152,222. As ContextMap produces unique mappings for each read, no further filtering was performed and all reads mapped to the human genome were used for downstream analyses. Number of mapped sequencing reads per genome position (= coverage, sum of 2 replicates) were visualized using the Integrative Genomics Viewer (IGV) [51]. No normalization was performed for this purpose.

### Quantification of gene expression and transcription read-through

Number of read fragments per gene were determined from the mapped 4sU-seq reads in a strand-specific manner using featureCounts [52] and gene annotations from Ensembl (version 87 for GRCh37). All read pairs (= fragments) overlapping exonic regions on the corresponding strand by ≥25bp were counted for the corresponding gene. HSV-1 gene annotations were obtained from GenBank (GenBank accession code: JN555585). Expression of cellular protein-coding and lincRNAs was quantified in terms of *fragments per kilobase of exons per million mapped reads* (FPKM) and averaged between replicates. Only reads mapped to the human genome were counted for the total number of mapped reads for FPKM calculation. Fold-changes in FPKM values were normalized by dividing by median fold-changes for housekeeping genes (as defined in [53]) to account for different levels of DoTT/DoG transcription and consequently different numbers of intergenic reads in different samples and conditions.

The percentage of transcription downstream or upstream of a gene (on the same strand) were calculated separately for each replicate as:

- %downstream transcription = 100 x (FPKM in 5kb downstream of gene)/(gene FPKM)
- %upstream transcription = 100 x (FPKM in 5kb upstream of gene)/(gene FPKM)

As both transcription downstream or upstream of the gene (= FPKM in 5kb downstream or upstream of gene) and transcription within the gene (= gene FPKM) are quantified in the same timeframe of infection using 4sU-seq, both should be affected to the same degree by a general decrease in transcription. Thus, calculation of this ratio cancels out the effect of any general decrease in transcription.

%downstream and upstream transcription were averaged between replicates and transcription read-through and read-in were then calculated as:

- read-through = %downstream transcription in infected or treated cells–%downstream transcription in uninfected or untreated cells
- read-in = %upstream transcription in infected or treated cells–%upstream transcription in uninfected or untreated cells.

If this resulted in negative values for a gene, read-through or read-in were set to 0. As calculation of %downstream transcription or %upstream transcription cancels out the effect of any overall decrease in transcription, calculation of read-through or read-in are independent of any such decrease.

Mock read-through values were calculated from the two replicates for each time-point for each condition. Here, *mock read-through(x,r)* = %downstream transcription in replicate r for sample x - %downstream transcription in replicate r’ for sample x. Here, $r,\;r^{'}\in \{1,2\}$ and $r^{'}\neq r$.

### Motif analysis

Occurrence numbers of all possible 6-mer nucleotide sequences were determined within 100nt up- or downstream of gene 3’ends. Spearman correlations between these counts for each gene and read-through values in each sample as well as significance of correlations were calculated using the *cor*.*test* function in R and adjusted for multiple testing for each sample using the method by Benjamini and Hochberg for controlling the false discovery rate (FDR) [54].

### Identification of induced splicing events

Splice junctions and read counts for splice junctions were determined from spliced 4sU-seq/RNA-seq read mappings. All predicted junctions were considered that used at least one annotated exon boundary and ended within the annotated 3’ and 5’ ends of the corresponding gene. Only reads were counted that included at least 5bp on either side of the splice junction. Regulation (up- or downregulation) of splice junctions was evaluated in terms of the odds-ratio: $\frac{\left(\frac{c_{j}^{*}}{c_{oj}^{*}}\right)}{\left(\frac{c_{j}}{c_{oj}}\right)}$. Here, $c_{j}^{*}$ and $c_{j}$ are the junction counts in infected or treated and uninfected or untreated cells, respectively. $c_{oj}^{*}$ and $c_{oj}$ are the counts for all other junctions of the same gene in infected or treated and uninfected or untreated cells, respectively. Odds-ratios and significance of odds-ratios were calculated from replicate data using the Mantel-Haenszel chi-squared test in R. Multiple testing correction was performed with the method by Benjamini and Hochberg [54]. Splicing events were considered significantly upregulated (downregulated) if the adjusted p-value was ≤ 0.01 and the odds-ratio ≥2 (≤0.5).

### Analysis of ATAC-seq data

Adapter trimming and mapping to human and HSV-1 genomes was performed as described for the 4sU-seq data. BAM files with mapped reads were converted to BED format using BEDTools [55] and OCRs were determined from these BED files using F-Seq with default parameters [56]. No filtering of OCRs was performed. Assignment of OCRs to gene promoters was performed using ChIPseeker [57]. 5kb dOCR length for each gene was calculated as the number of nucleotides in the 5kb directly downstream of the gene 3’ end that overlap an OCR. dOCR length for a gene was calculated as the total genomic length of downstream OCRs (including only the positions downstream of the gene 3’ end) assigned to the gene in the following way. First, all OCRs overlapping with the 10kb downstream of a gene were assigned to this gene. Second, OCRs starting at most 5kb downstream of the so far most downstream OCR of a gene were also assigned to this gene. This was performed iteratively, until no more OCRs could be assigned. 5kb dOCR length and dOCR length were averaged between replicates. Empirical cumulative distribution functions for dOCR length were calculated with the ecdf function in R [58]. For this purpose, genes were grouped according to read-through at 7-8h p.i. HSV-1 infection or salt or heat stress. Thresholds were chosen such that genes without DoTT (read-through ≤5%) were in one group and the remaining genes were divided in equal-sized groups according to read-through.

### Analysis of histone modification marks downstream of genes

Narrow peaks for ChIP-seq data of histone modification marks (H3K27ac, H3K27me3, H3K36me3, H3K4me1, H3K4me3, H3K9me3) in HFF were downloaded from ENCODE (epigenome series ENCSR403RCR). Presence of histone modification marks downstream of each gene was evaluated by determining the number of nucleotides in the 5kb directly downstream of the gene 3’ end that overlap peaks for the corresponding histone marks (denoted as downstream histone mark length). Spearman correlations between read-through in all conditions and downstream histone mark length and significance of correlations were calculated using the *cor*.*test* function in R and adjusted for multiple testing for each sample using the method by Benjamini and Hochberg [54].

### Accession numbers

The datasets generated and analyzed in the current study are available in the Gene Expression Omnibus (GEO) database under the following accession numbers:

4sU-seq data of HSV-1 infection: GSE59717.

4sU-seq data of salt and heat stress: GSE100469.

RNA-seq of total, cytoplasmic, nucleoplasmic and chromatin-associated RNA: GSE100576.

ATAC-seq data for HSV-1 infected cells: GSE100611.

ATAC-seq data for salt and heat stress: GSE101731.

## Supporting information

S1 File Supplementary tables. Table A provides read-through values obtained from 4sU-seq data for HSV-1 infection, salt and heat stress. Table B provides read-through values for total, cytoplasmic, nucleoplasmic and chromatin-associated RNA at 8h p.i. HSV-1 infection. Table C lists genes for which read-through transcripts at 8h p.i. HSV-1 infection appear to remain at the chromatin, i.e. genes with ≤5% read-through in nucleoplasmic and cytoplasmic RNA, but ≥25% in chromatin-associated RNA. Table D lists all qRT-PCR primers used in this study.
(XLSX)

S2 File Supplementary methods. Protocol for the separation of subcellular RNA fractions used in this study.
(PDF)

S3 File Supplementary figures. Contains Supplementary Figures A-R and legends.
(PDF)

## References

[1] Porrua O, Libri D. Transcription termination and the control of the transcriptome: why, where and how to stop. Nat Rev Mol Cell Biol. 2015;16(3):190–202. doi: 10.1038/nrm3943 25650800 10.1038/nrm3943

[2] Proudfoot NJ. Transcriptional termination in mammals: Stopping the RNA polymerase II juggernaut. Science. 2016;352(6291):aad9926 doi: 10.1126/science.aad9926 ; PubMed Central PMCID: PMCPMC5144996. 27284201 10.1126/science.aad9926 PMC5144996

[3] Kwong AD, Frenkel N. Herpes simplex virus-infected cells contain a function(s) that destabilizes both host and viral mRNAs. Proc Natl Acad Sci U S A. 1987;84(7):1926–30. Epub 1987/04/01. ; PubMed Central PMCID: PMC304554. 3031658 10.1073/pnas.84.7.1926 PMC304554

[4] Oroskar AA, Read GS. Control of mRNA stability by the virion host shutoff function of herpes simplex virus. J Virol. 1989;63(5):1897–906. Epub 1989/05/01. ; PubMed Central PMCID: PMC250601. 2539493 10.1128/jvi.63.5.1897-1906.1989 PMC250601

[5] Sandri-Goldin RM. The many roles of the regulatory protein ICP27 during herpes simplex virus infection. Front Biosci. 2008;13:5241–56. Epub 2008/05/30. doi: https://doi.org/10.2741/3078 [pii]. 18508584 10.2741/3078

[6] Sandri-Goldin RM. The many roles of the highly interactive HSV protein ICP27, a key regulator of infection. Future Microbiol. 2011;6(11):1261–77. Epub 2011/11/16. doi: 10.2217/fmb.11.119 22082288 10.2217/fmb.11.119

[7] Smith RW, Malik P, Clements JB. The herpes simplex virus ICP27 protein: a multifunctional post-transcriptional regulator of gene expression. Biochem Soc Trans. 2005;33(Pt 3):499–501. Epub 2005/05/27. doi: BST0330499 [pii] doi: 10.1042/BST0330499 15916551 10.1042/BST0330499

[8] Fraser KA, Rice SA. Herpes simplex virus immediate-early protein ICP22 triggers loss of serine 2-phosphorylated RNA polymerase II. J Virol. 2007;81(10):5091–101. doi: 10.1128/JVI.00184-07 ; PubMed Central PMCID: PMC1900222. 17344289 10.1128/JVI.00184-07 PMC1900222

[9] Ou M, Sandri-Goldin RM. Inhibition of cdk9 during herpes simplex virus 1 infection impedes viral transcription. PLoS One. 2013;8(10):e79007 Epub 2013/11/10. doi: 10.1371/journal.pone.0079007 [pii]. ; PubMed Central PMCID: PMC3799718. 24205359 10.1371/journal.pone.0079007 PMC3799718

[10] Rutkowski AJ, Erhard F, L'Hernault A, Bonfert T, Schilhabel M, Crump C, et al Widespread disruption of host transcription termination in HSV-1 infection. Nature communications. 2015;6:7126 doi: 10.1038/ncomms8126 ; PubMed Central PMCID: PMC4441252. 25989971 10.1038/ncomms8126 PMC4441252

[11] Vilborg A, Passarelli MC, Yario TA, Tycowski KT, Steitz JA. Widespread Inducible Transcription Downstream of Human Genes. Mol Cell. 2015;59(3):449–61. doi: 10.1016/j.molcel.2015.06.016 ; PubMed Central PMCID: PMC4530028. 26190259 10.1016/j.molcel.2015.06.016 PMC4530028

[12] Grosso AR, Leite AP, Carvalho S, Matos MR, Martins FB, Vitor AC, et al Pervasive transcription read-through promotes aberrant expression of oncogenes and RNA chimeras in renal carcinoma. Elife. 2015;4 doi: 10.7554/eLife.09214 26575290 10.7554/eLife.09214 PMC4744188

[13] Vilborg A, Sabath N, Wiesel Y, Nathans J, Levy-Adam F, Yario TA, et al Comparative analysis reveals genomic features of stress-induced transcriptional readthrough. Proc Natl Acad Sci U S A. 2017;114(40):E8362–E8371. doi: 10.1073/pnas.1711120114 28928151 10.1073/pnas.1711120114 PMC5635911

[14] Wyler E, Menegatti J, Franke V, Kocks C, Boltengagen A, Hennig T, et al Widespread activation of antisense transcription of the host genome during herpes simplex virus 1 infection. Genome Biol. 2017;18(1):209 doi: 10.1186/s13059-017-1329-5 ; PubMed Central PMCID: PMCPMC5663069. 29089033 10.1186/s13059-017-1329-5 PMC5663069

[15] Buenrostro JD, Giresi PG, Zaba LC, Chang HY, Greenleaf WJ. Transposition of native chromatin for fast and sensitive epigenomic profiling of open chromatin, DNA-binding proteins and nucleosome position. Nat Methods. 2013;10(12):1213–8. Epub 2013/10/08. doi: 10.1038/nmeth.2688 [pii]. ; PubMed Central PMCID: PMC3959825. 24097267 10.1038/nmeth.2688 PMC3959825

[16] Friedel CC, Dölken L. Metabolic tagging and purification of nascent RNA: implications for transcriptomics. Mol Biosyst. 2009;5(11):1271–8. doi: 10.1039/b911233b 19823741 10.1039/b911233b

[17] Taddeo B, Esclatine A, Roizman B. The patterns of accumulation of cellular RNAs in cells infected with a wild-type and a mutant herpes simplex virus 1 lacking the virion host shutoff gene. Proc Natl Acad Sci U S A. 2002;99(26):17031–6. Epub 2002/12/14. doi: 10.1073/pnas.252588599 [pii]. ; PubMed Central PMCID: PMC139264. 12481033 10.1073/pnas.252588599 PMC139264

[18] Ray D, Kazan H, Cook KB, Weirauch MT, Najafabadi HS, Li X, et al A compendium of RNA-binding motifs for decoding gene regulation. Nature. 2013;499(7457):172–7. doi: 10.1038/nature12311 ; PubMed Central PMCID: PMCPMC3929597. 23846655 10.1038/nature12311 PMC3929597

[19] Gruber AJ, Schmidt R, Gruber AR, Martin G, Ghosh S, Belmadani M, et al A comprehensive analysis of 3' end sequencing data sets reveals novel polyadenylation signals and the repressive role of heterogeneous ribonucleoprotein C on cleavage and polyadenylation. Genome Res. 2016;26(8):1145–59. doi: 10.1101/gr.202432.115 ; PubMed Central PMCID: PMCPMC4971764. 27382025 10.1101/gr.202432.115 PMC4971764

[20] Huang DW, Sherman BT, Lempicki RA. Systematic and integrative analysis of large gene lists using DAVID bioinformatics resources. Nature protocols. 2009;4(1):44–57. Epub 2009/01/10. doi: 10.1038/nprot.2008.211 19131956 10.1038/nprot.2008.211

[21] Kimura T, Nakayama K, Penninger J, Kitagawa M, Harada H, Matsuyama T, et al Involvement of the IRF-1 transcription factor in antiviral responses to interferons. Science. 1994;264(5167):1921–4. 8009222 10.1126/science.8009222

[22] Dutia BM, Allen DJ, Dyson H, Nash AA. Type I interferons and IRF-1 play a critical role in the control of a gammaherpesvirus infection. Virology. 1999;261(2):173–9. doi: 10.1006/viro.1999.9834 10497103 10.1006/viro.1999.9834

[23] Brien JD, Daffis S, Lazear HM, Cho H, Suthar MS, Gale M Jr, et al Interferon regulatory factor-1 (IRF-1) shapes both innate and CD8(+) T cell immune responses against West Nile virus infection. PLoS Pathog. 2011;7(9):e1002230 doi: 10.1371/journal.ppat.1002230 ; PubMed Central PMCID: PMCPMC3164650. 21909274 10.1371/journal.ppat.1002230 PMC3164650

[24] Ru J, Sun H, Fan H, Wang C, Li Y, Liu M, et al MiR-23a facilitates the replication of HSV-1 through the suppression of interferon regulatory factor 1. PLoS One. 2014;9(12):e114021 doi: 10.1371/journal.pone.0114021 ; PubMed Central PMCID: PMCPMC4252059. 25461762 10.1371/journal.pone.0114021 PMC4252059

[25] Cheshenko N, Del Rosario B, Woda C, Marcellino D, Satlin LM, Herold BC. Herpes simplex virus triggers activation of calcium-signaling pathways. J Cell Biol. 2003;163(2):283–93. doi: 10.1083/jcb.200301084 ; PubMed Central PMCID: PMCPMC2173509. 14568989 10.1083/jcb.200301084 PMC2173509

[26] Cheshenko N, Liu W, Satlin LM, Herold BC. Multiple receptor interactions trigger release of membrane and intracellular calcium stores critical for herpes simplex virus entry. Mol Biol Cell. 2007;18(8):3119–30. doi: 10.1091/mbc.E07-01-0062 ; PubMed Central PMCID: PMCPMC1949381. 17553929 10.1091/mbc.E07-01-0062 PMC1949381

[27] Kalamvoki M, Roizman B. Bcl-2 blocks accretion or depletion of stored calcium but has no effect on the redistribution of IP3 receptor I mediated by glycoprotein E of herpes simplex virus 1. J Virol. 2007;81(12):6316–25. doi: 10.1128/JVI.00311-07 ; PubMed Central PMCID: PMC1900130. 17409148 10.1128/JVI.00311-07 PMC1900130

[28] Tsien RY. New calcium indicators and buffers with high selectivity against magnesium and protons: design, synthesis, and properties of prototype structures. Biochemistry. 1980;19(11):2396–404. 6770893 10.1021/bi00552a018

[29] Rosner M, Schipany K, Hengstschlager M. Merging high-quality biochemical fractionation with a refined flow cytometry approach to monitor nucleocytoplasmic protein expression throughout the unperturbed mammalian cell cycle. Nature protocols. 2013;8(3):602–26. doi: 10.1038/nprot.2013.011 23449254 10.1038/nprot.2013.011

[30] Pandya-Jones A, Black DL. Co-transcriptional splicing of constitutive and alternative exons. RNA. 2009;15(10):1896–908. doi: 10.1261/rna.1714509 ; PubMed Central PMCID: PMCPMC2743041. 19656867 10.1261/rna.1714509 PMC2743041

[31] Proudfoot NJ, Furger A, Dye MJ. Integrating mRNA processing with transcription. Cell. 2002;108(4):501–12. 11909521 10.1016/s0092-8674(02)00617-7

[32] Cramer P, Caceres JF, Cazalla D, Kadener S, Muro AF, Baralle FE, et al Coupling of transcription with alternative splicing: RNA pol II promoters modulate SF2/ASF and 9G8 effects on an exonic splicing enhancer. Mol Cell. 1999;4(2):251–8. 10488340 10.1016/s1097-2765(00)80372-x

[33] Read GS. Virus-encoded endonucleases: expected and novel functions. Wiley Interdiscip Rev RNA. 2013;4(6):693–708. Epub 2013/08/01. doi: 10.1002/wrna.1188 23900973 10.1002/wrna.1188

[34] Rabani M, Levin JZ, Fan L, Adiconis X, Raychowdhury R, Garber M, et al Metabolic labeling of RNA uncovers principles of RNA production and degradation dynamics in mammalian cells. Nat Biotechnol. 2011;29(5):436–42. doi: 10.1038/nbt.1861 ; PubMed Central PMCID: PMC3114636. 21516085 10.1038/nbt.1861 PMC3114636

[35] Davari K, Lichti J, Gallus C, Greulich F, Uhlenhaut NH, Heinig M, et al Rapid Genome-wide Recruitment of RNA Polymerase II Drives Transcription, Splicing, and Translation Events during T Cell Responses. Cell Rep. 2017;19(3):643–54. doi: 10.1016/j.celrep.2017.03.069 28423325 10.1016/j.celrep.2017.03.069

[36] Marcinowski L, Lidschreiber M, Windhager L, Rieder M, Bosse JB, Radle B, et al Real-time Transcriptional Profiling of Cellular and Viral Gene Expression during Lytic Cytomegalovirus Infection. PLoS Pathog. 2012;8(9):e1002908 Epub 2012/09/13. doi: 10.1371/journal.ppat.1002908 [pii]. ; PubMed Central PMCID: PMC3435240. 22969428 10.1371/journal.ppat.1002908 PMC3435240

[37] Venkatesh S, Workman JL. Histone exchange, chromatin structure and the regulation of transcription. Nat Rev Mol Cell Biol. 2015;16(3):178–89. doi: 10.1038/nrm3941 25650798 10.1038/nrm3941

[38] Conn KL, Hendzel MJ, Schang LM. Linker histones are mobilized during infection with herpes simplex virus type 1. J Virol. 2008;82(17):8629–46. doi: 10.1128/JVI.00616-08 ; PubMed Central PMCID: PMCPMC2519646. 18579611 10.1128/JVI.00616-08 PMC2519646

[39] Conn KL, Hendzel MJ, Schang LM. The differential mobilization of histones H3.1 and H3.3 by herpes simplex virus 1 relates histone dynamics to the assembly of viral chromatin. PLoS Pathog. 2013;9(10):e1003695 doi: 10.1371/journal.ppat.1003695 ; PubMed Central PMCID: PMCPMC3795045. 24130491 10.1371/journal.ppat.1003695 PMC3795045

[40] Yager DR, Bachenheimer SL. Synthesis and metabolism of cellular transcripts in HSV-1 infected cells. Virus Genes. 1988;1(2):135–48. 2853485 10.1007/BF00555933

[41] Schek N, Bachenheimer SL. Degradation of cellular mRNAs induced by a virion-associated factor during herpes simplex virus infection of Vero cells. J Virol. 1985;55(3):601–10. ; PubMed Central PMCID: PMCPMC255019. 4020960 10.1128/jvi.55.3.601-610.1985 PMC255019

[42] Duina AA. Histone Chaperones Spt6 and FACT: Similarities and Differences in Modes of Action at Transcribed Genes. Genet Res Int. 2011;2011:625210 doi: 10.4061/2011/625210 ; PubMed Central PMCID: PMCPMC3335715. 22567361 10.4061/2011/625210 PMC3335715

[43] Mayer A, Heidemann M, Lidschreiber M, Schreieck A, Sun M, Hintermair C, et al CTD tyrosine phosphorylation impairs termination factor recruitment to RNA polymerase II. Science. 2012;336(6089):1723–5. doi: 10.1126/science.1219651 22745433 10.1126/science.1219651

[44] Burugula BB, Jeronimo C, Pathak R, Jones JW, Robert F, Govind CK. Histone deacetylases and phosphorylated polymerase II C-terminal domain recruit Spt6 for cotranscriptional histone reassembly. Mol Cell Biol. 2014;34(22):4115–29. doi: 10.1128/MCB.00695-14 ; PubMed Central PMCID: PMCPMC4248711. 25182531 10.1128/MCB.00695-14 PMC4248711

[45] Eick D, Geyer M. The RNA polymerase II carboxy-terminal domain (CTD) code. Chem Rev. 2013;113(11):8456–90. doi: 10.1021/cr400071f 23952966 10.1021/cr400071f

[46] Fox HL, Dembowski JA, DeLuca NA. A Herpesviral Immediate Early Protein Promotes Transcription Elongation of Viral Transcripts. MBio. 2017;8(3). doi: 10.1128/mBio.00745-17 ; PubMed Central PMCID: PMCPMC5472187. 28611249 10.1128/mBio.00745-17 PMC5472187

[47] Dölken L, Ruzsics Z, Radle B, Friedel CC, Zimmer R, Mages J, et al High-resolution gene expression profiling for simultaneous kinetic parameter analysis of RNA synthesis and decay. RNA. 2008;14(9):1959–72. doi: 10.1261/rna.1136108 18658122 10.1261/rna.1136108 PMC2525961

[48] Martin M. Cutadapt removes adapter sequences from high-throughput sequencing reads. EMBnet.Journal. 2011;17(1):10–12. doi: 10.14806/ej.17.1.200pp. 10–12.

[49] Bonfert T, Kirner E, Csaba G, Zimmer R, Friedel CC. ContextMap 2: fast and accurate context-based RNA-seq mapping. BMC Bioinformatics. 2015;16:122 doi: 10.1186/s12859-015-0557-5 ; PubMed Central PMCID: PMCPMC4411664. 25928589 10.1186/s12859-015-0557-5 PMC4411664

[50] Li H, Durbin R. Fast and accurate short read alignment with Burrows-Wheeler transform. Bioinformatics. 2009;25(14):1754–60. Epub 2009/05/20. doi: 10.1093/bioinformatics/btp324 [pii]. ; PubMed Central PMCID: PMC2705234. 19451168 10.1093/bioinformatics/btp324 PMC2705234

[51] Robinson JT, Thorvaldsdottir H, Winckler W, Guttman M, Lander ES, Getz G, et al Integrative genomics viewer. Nat Biotechnol. 2011;29(1):24–6. Epub 2011/01/12. doi: 10.1038/nbt.1754 ; PubMed Central PMCID: PMC3346182. 21221095 10.1038/nbt.1754 PMC3346182

[52] Liao Y, Smyth GK, Shi W. featureCounts: an efficient general purpose program for assigning sequence reads to genomic features. Bioinformatics. 2014;30(7):923–30. doi: 10.1093/bioinformatics/btt656 24227677 10.1093/bioinformatics/btt656

[53] Eisenberg E, Levanon EY. Human housekeeping genes, revisited. Trends Genet. 2013;29(10):569–74. doi: 10.1016/j.tig.2013.05.010 23810203 10.1016/j.tig.2013.05.010

[54] Benjamini Y, Hochberg Y. Controlling the false discovery rate: a practical and powerful approach to multiple testing. Journal of the Royal Statistical Society. Series B (Methodological) 1995; 57: 289–300.

[55] Quinlan AR, Hall IM. BEDTools: a flexible suite of utilities for comparing genomic features. Bioinformatics. 2010;26(6):841–2. doi: 10.1093/bioinformatics/btq033 ; PubMed Central PMCID: PMCPMC2832824. 20110278 10.1093/bioinformatics/btq033 PMC2832824

[56] Boyle AP, Guinney J, Crawford GE, Furey TS. F-Seq: a feature density estimator for high-throughput sequence tags. Bioinformatics. 2008;24(21):2537–8. doi: 10.1093/bioinformatics/btn480 ; PubMed Central PMCID: PMCPMC2732284. 18784119 10.1093/bioinformatics/btn480 PMC2732284

[57] Yu G, Wang LG, He QY. ChIPseeker: an R/Bioconductor package for ChIP peak annotation, comparison and visualization. Bioinformatics. 2015;31(14):2382–3. doi: 10.1093/bioinformatics/btv145 25765347 10.1093/bioinformatics/btv145

[58] R Core Team. R: A language and environment for statistical computing. Vienna, Austria: R Foundation for Statistical Computing; 2016.

