# Supplementary material for: HSV-1-induced disruption of transcription termination resembles a cellular stress response but selectively increases chromatin accessibility downstream of genes
Source: PLoS Pathog. 2018 Mar 26;14(3):e1006954. doi: 10.1371/journal.ppat.1006954 (PMC5886697; doi:10.1371/journal.ppat.1006954)
Supplement: S2 File — Protocol for the separation of subcellular RNA fractions used in this study. (PDF) [file ppat.1006954.s002.pdf]

Supplementary Methods

Fractionation of cytoplasmic, nucleoplasmic and chromatin-bound RNA

Adapted from:

- Rosner M, Schipany K, Hengstschlager M: Merging high-quality biochemical fractionation with a refined flow cytometry approach to monitor nucleocytoplasmic protein expression throughout the unperturbed mammalian cell cycle. Nat Protoc 2013, 8:602-626.
- Pandya-Jones A, Black DL: Co-transcriptional splicing of constitutive and alternative exons. RNA 2009, 15:1896-1908.

Materials:

- PBS with 1mM EDTA (PBSE)
- Proteinase K
- 10% IGEPAL CA-630
- Liquid nitrgen

- **Buffer A (cytoplasm/nuclei separation)**
  20mM Tris, pH 7.6
  0.1mM EDTA
  2mM $MgCl_2$

- **Buffer A-630**
  Buffer A
  1% IGEPAL CA-630

- **Glycerol buffer**
  20 mM Tris-HCl (pH 7.9)
  75 mM NaCl
  0.5 mM EDTA
  0.85 mM DTT
  50% glycerol

- **Nuclei lysis buffer**
  10 mM HEPES (pH 7.6)
  1 mM DTT
  7.5 mM $MgCl_2$
  0.2 mM EDTA
  0.3 M NaCl
  1 M UREA
  1% NP-40

- **2 x RIPA buffer**
  0.1M Tris pH 7.5
  0.3M NaCl
  0.2% SDS
  1% Na deoxycholate
  2% Triton X-100
  ➢ *Before use, add protease inhibitors*
  ➢ *Add benzonase*

Protocol:

(Optimized for ~6 x 10^6 HFFFs*)*

1. Wash cells with ice-cold PBSE and aspirate it thoroughly
2. Add 1mL of ice-cold PBSE, scrape the cells and collect in a 1.5mL tube.
3. Pellet the cells (20s, 3000 x g, 4°C) and place on ice.
4. Resuspend in 450µL Buffer A and pipette several times.
   ➢ *Mix 50µL with 50µL 2x RIPA buffer for protein analysis, add 1µL benzonase.*
   ➢ *Collect 50µL and add 1mL Trizol for total RNA isolation.*
5. Incubate 2 min at room temperature and 10 minutes on ice.
6. Add 35µL 10% IGEPAL-630 and pipette several times.
7. Pellet nuclei at 3000 x g, 30s, 4°C.
8. Collect 300µL of the supernatant (cytoplasmic fraction) and keep on ice.
   ➢ *Mix 50µL with 50µL 2x RIPA buffer for protein analysis.*
9. Carefully remove and discard the rest of the supernatant.
10. Add 300µL of buffer A-630 and pipette several times
11. Pellet the nuclei at 3000 x g, 30s, 4°C, remove supernatant.
12. Repeat steps 10 and 11 twice.
13. Resuspend in 150µL ice-cold glycerol buffer
14. Add 150µL ice-cold nuclei lysis buffer.
15. Vortex for a few seconds.
16. Snap-freeze in liquid nitrogen twice and incubate on ice for 25 minutes.

17. Centrifuge for 20 minutes at 4°C at max speed.
18. Collect the supernatant (nucleoplasmic fraction)
    - *Mix 50µL with 50µL 2x RIPA buffer for protein analysis.*
19. Treat the remaining 250µL and the cytosolic fraction from step 8 with proteinase K (5µL, 37°C, 1 hour).
20. Resuspend the pellet in 500µL PBSE.
    - *Mix 50µL with 50µL 2x RIPA buffer for protein analysis, add 1µL benzonase.*
21. Pellet the chromatin at 20,000 x g, 10 min, 4°C.
22. Lyse with 1mL Trizol.
23. Add 750 Trizol LS to the proteinase K-digested fractions.
24. Isolate RNA from all four fractions using the sodium citrate protocol and glycogen for precipitation.
