# Supplementary material for: HSV-1-induced disruption of transcription termination resembles a cellular stress response but selectively increases chromatin accessibility downstream of genes
Source: PLoS Pathog. 2018 Mar 26;14(3):e1006954. doi: 10.1371/journal.ppat.1006954 (PMC5886697; doi:10.1371/journal.ppat.1006954)
Supplement: S3 File — Contains Supplementary Figures A-R and legends. (PDF) [file ppat.1006954.s003.pdf]

# Supplementary Figures

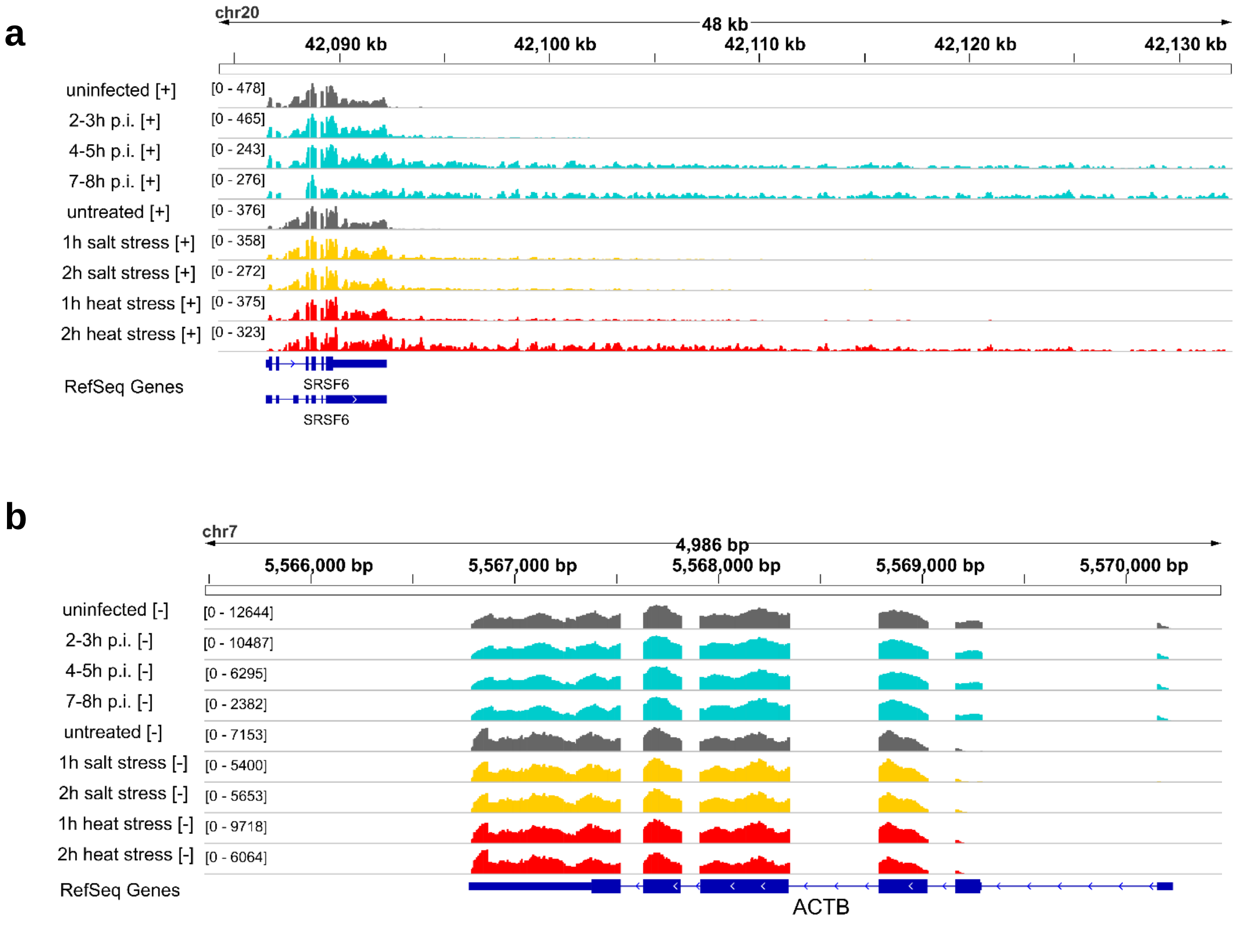


**Figure A: Read distribution downstream of example genes**
(a-b) 4sU-seq read coverage for the genes SRSF6 (a) and ACTB (b) in uninfected/untreated samples (gray), during HSV-1 infection (cyan) and in salt (yellow) and heat (red) stress. While SRSF6 shows extensive DoTT/DoG transcription, ACTB is completely spared from this effect. Only reads mapping to the corresponding strand are shown. Read coverage ranges are shown in square brackets on the y-axis. RefSeq gene annotation is indicated below (blue). Boxes indicate coding regions and untranslated regions (UTRs; narrow boxes) and lines intronic regions. The transcribed strand is indicated by the direction of the arrowheads.
Links to UCSC genome browser sessions showing read coverage for all genes and samples separately for both replicates can be found at www.bio.ifi.lmu.de/HSV-1. Links for individual genes are also included in Table A in S1 File.

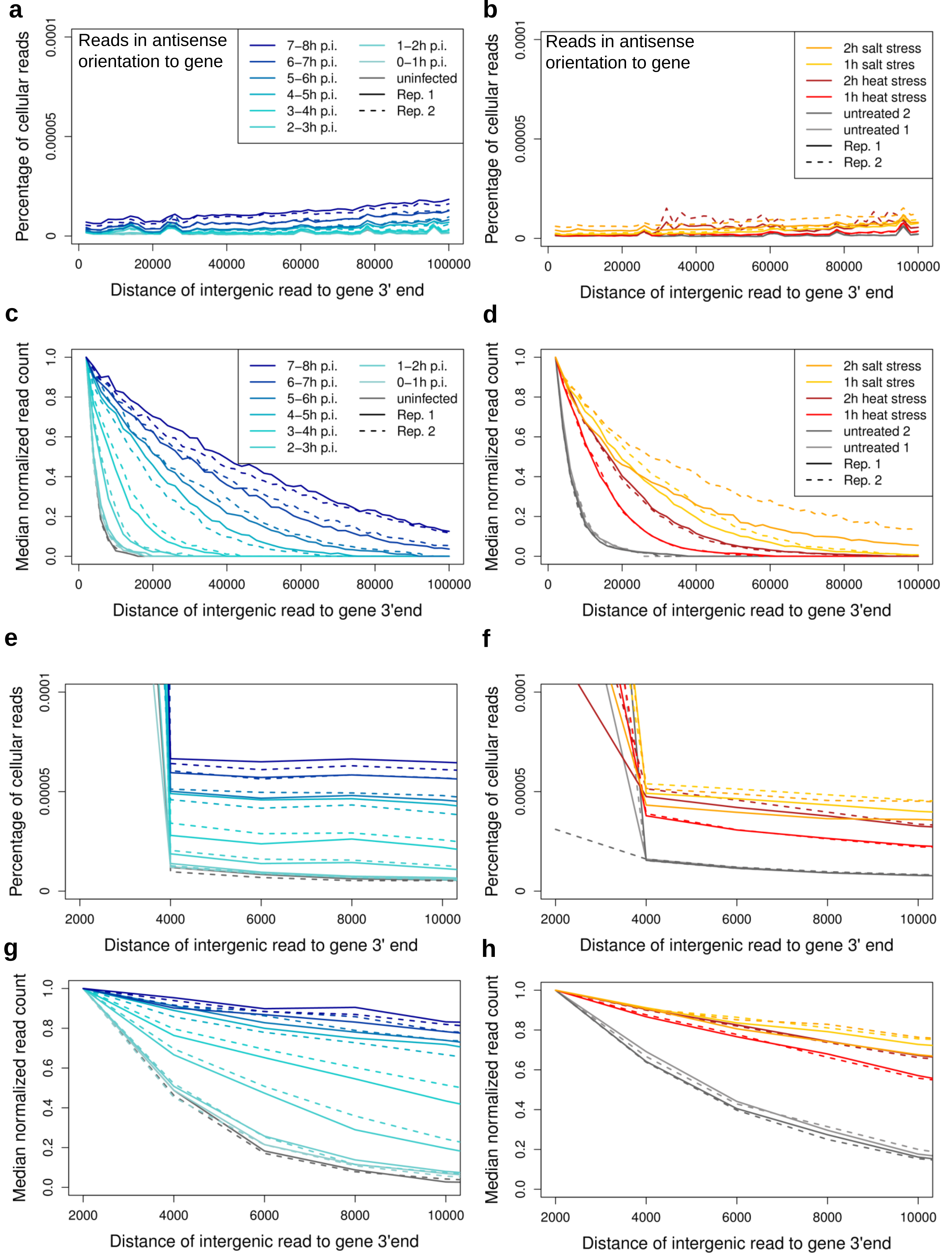


**Figure B: Legend on next page**

**Figure B: Read distribution downstream of genes**

(a-b) Distribution of reads mapping in *antisense* direction downstream of annotated gene 3' ends in HSV-1 infection (a) and salt and heat stress (b) (shown separately for the two replicates: solid lines=replicate 1, dashed lines=replicate 2). Only gene 3' ends with no gene on either strand within the 100kb downstream region were considered. Read counts in *antisense* direction to the gene were determined in 2kb windows downstream of gene 3' ends and divided by window length and the total number of mapped reads. This shows no peak in read counts directly downstream of genes in contrast to reads mapping on the sense strand (Figure 1e,f in main manuscript). The slight increase of antisense reads with increasing distance from gene 3' ends reflects read-through transcription of genes on the opposite strand outside of the 100kb window.

(c-d) Median per-gene distribution of reads mapping in *sense* direction downstream of annotated gene 3' ends in HSV-1 infection (c) and salt and heat stress (d). For this purpose, reads on the *sense* strand were counted for individual genes in 2kb windows downstream of gene 3' ends. Here, only genes were considered if there was no downstream gene on either strand within 100kb of the gene 3' end. Read counts for each gene were first divided by window length and then divided by the values for the first 2kb window directly downstream of the gene. Curves indicate median values over all genes considered. Results for the two replicates are shown separately (solid lines=replicate 1, dashed lines=replicate 2).

(e,f) Figure 1e and f from the main manuscript restricted to the first 10kb. Values on the y-axis were plotted against the end of the 2kb windows on the x-axis.

(g,h) Figures B(c,d) restricted to the first 10kb.

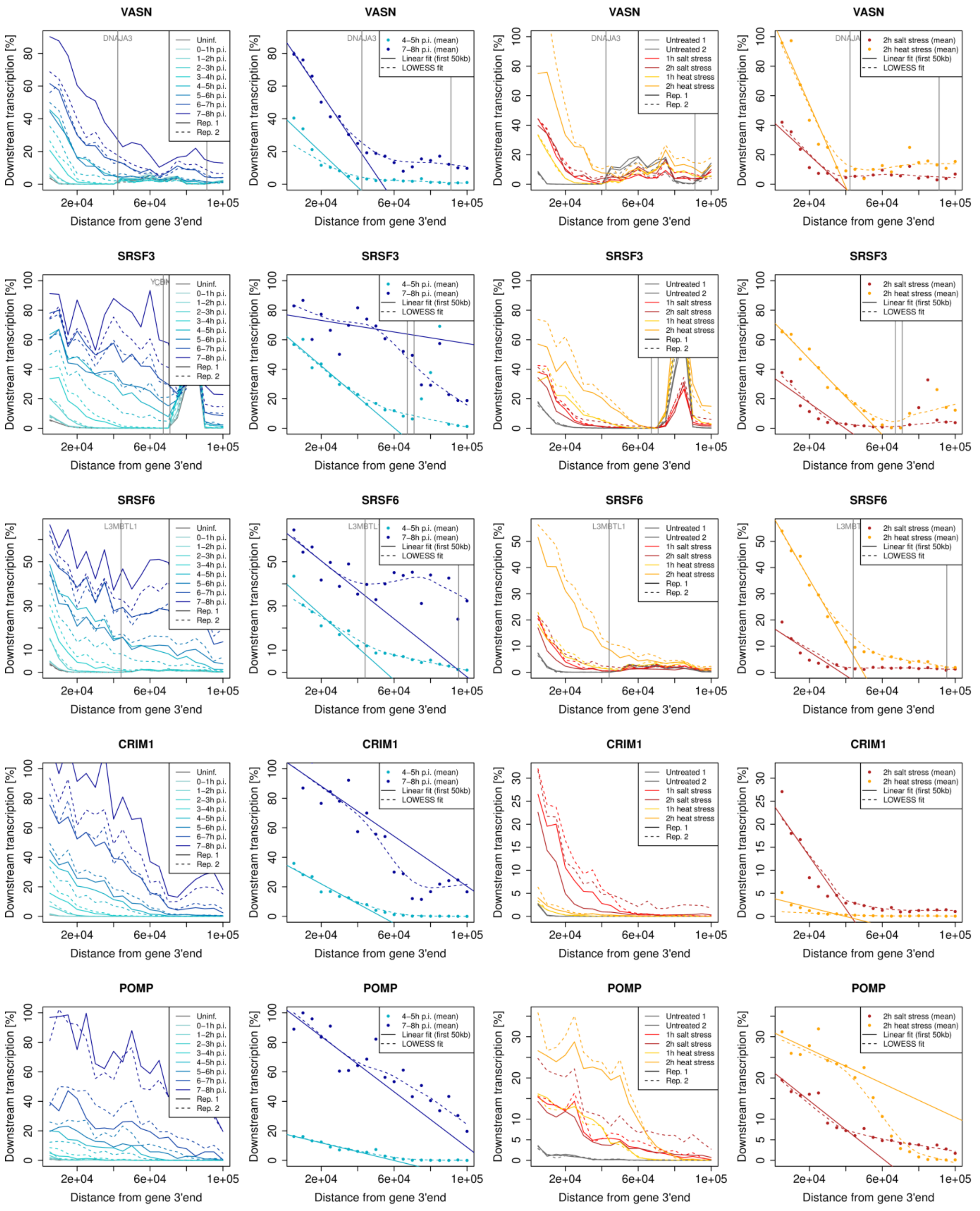


**Figure C: Downstream transcription for individual genes**

Downstream transcription [in %] in 5kb windows for the first 100kb downstream of the gene 3' ends for 5 example genes with read-through transcription in HSV-1 infection (first column) and salt and heat stress (third column). Downstream transcription for each window was calculated as described in the methods section for the first 5kb window. Gray vertical lines indicate 5' ends of downstream genes on the same strand. The second and fourth column show linear and LOWESS (locally weighted scatterplot smoothing) fits on replicate averages for selected time-points in HSV-1 infection (second column) and 2h salt and heat stress (fourth column), respectively. The linear fit was performed for the first 50kb or the region up to the next downstream gene if it was closer than 50kb.

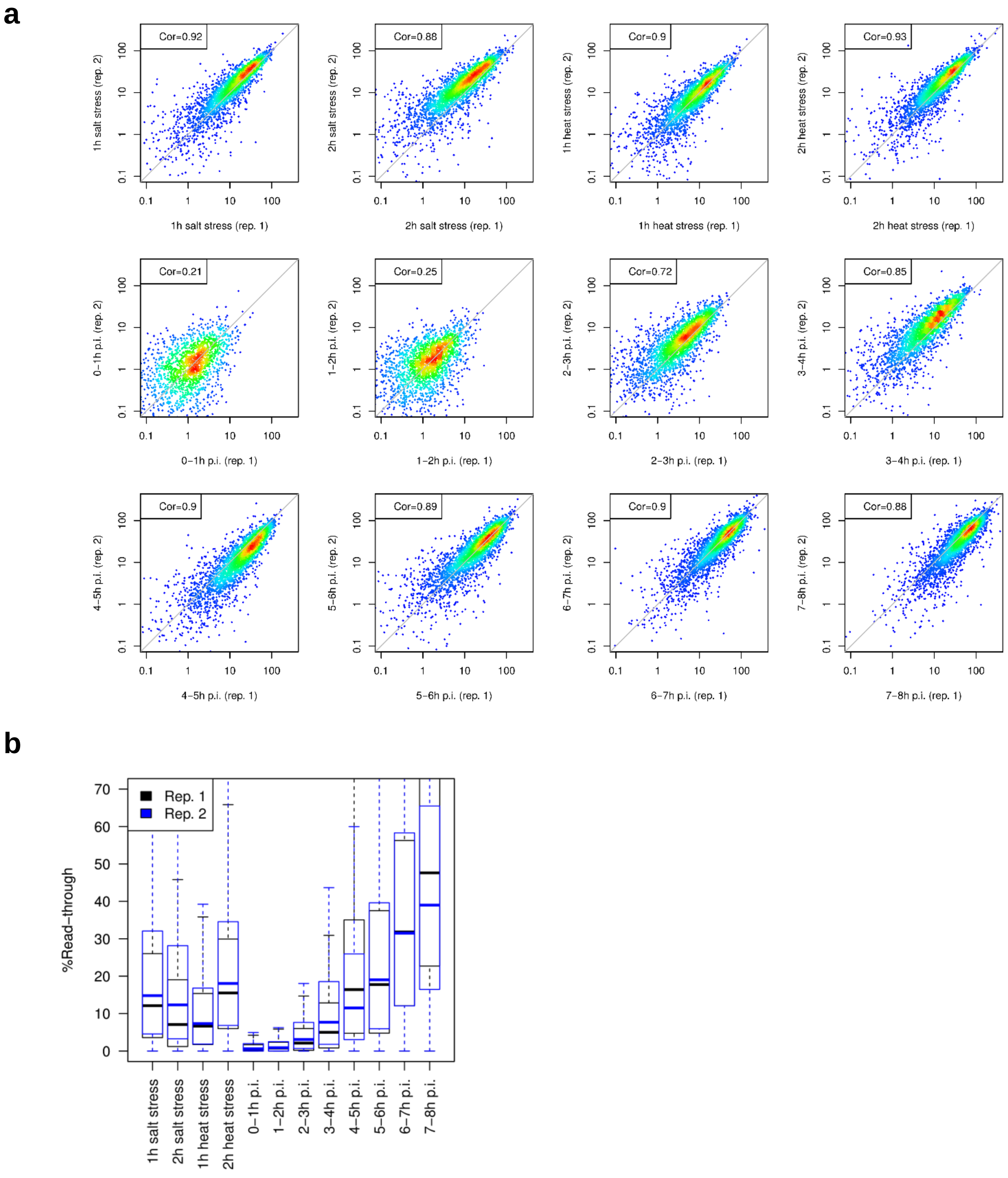


**Figure D: Reproducibility of read-through between replicates**
(a) Scatterplots comparing read-through between the two replicates. Colors indicate density of points (red=highest density, blue=lowest density.). Spearman correlation (Cor) is also indicated. (b) Boxplots showing the distribution of read-through in salt and heat stress and HSV-1 infection separately for replicate 1 (black) and 2 (blue).

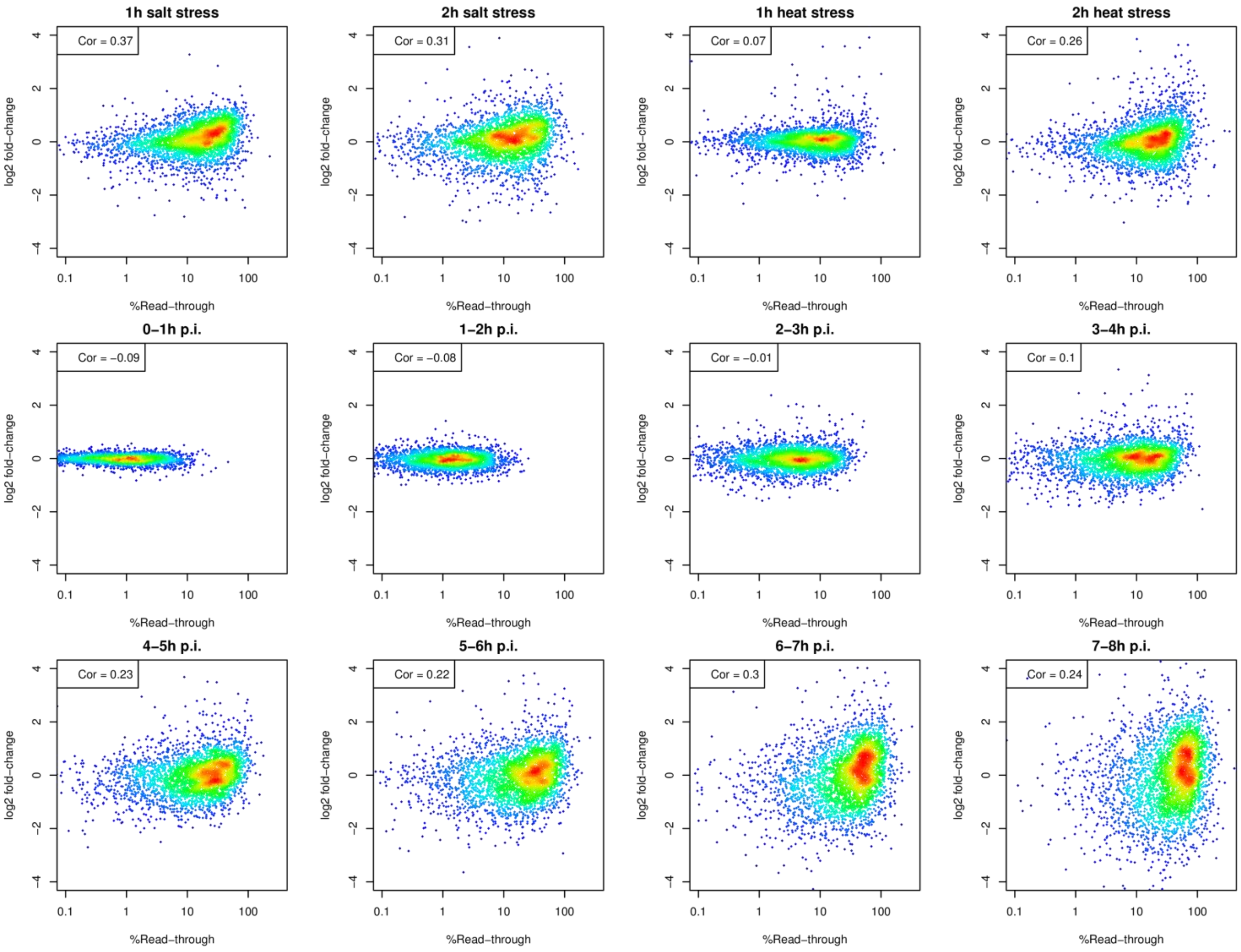


**Figure E: Correlation between read-through and gene expression fold-changes**
Scatterplots of read-through values against log2 gene expression fold-changes for individual genes for all samples. Colors indicate density of points (red=highest density, blue=lowest density.) Fold-changes were normalized by dividing by median fold-changes of housekeeping genes defined by Eisenberg and Levanon (see Materials and Methods in main manuscript). Figures also show the Spearman correlation (Cor), which is independent of normalization.

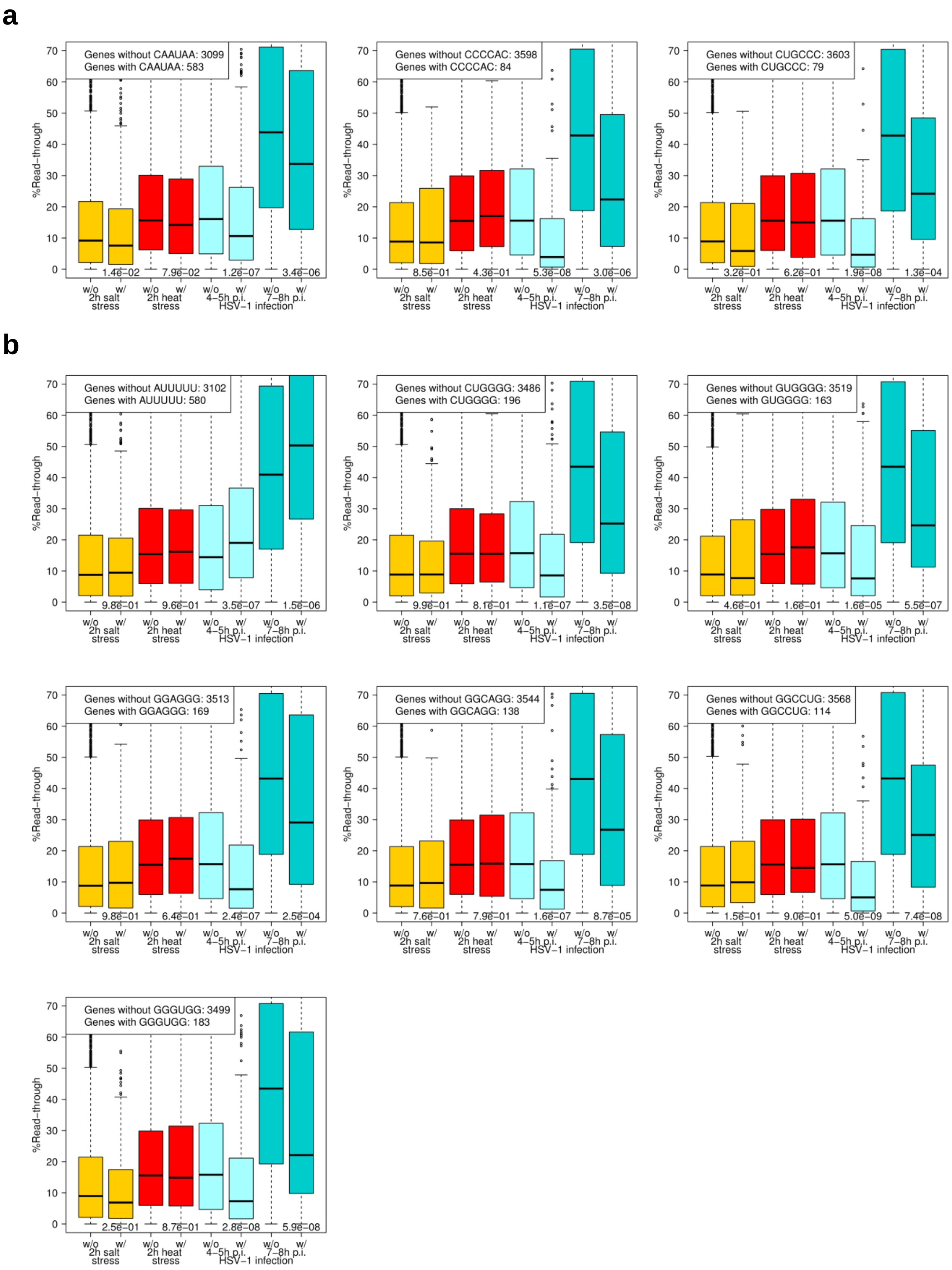


**Figure F: Correlation of read-through and presence of sequence motifs**
(a-b) Boxplots showing the distribution of read-through in 2h salt and heat stress and 4-5h and 7-8h p.i. for genes without (w/o) or with (w/) at least one occurrence of certain motifs in the 100nt upstream (a) or downstream (b) of gene 3’ ends. Results are shown for all motifs with significant correlations (see main Figure  2c). P-values of Wilcoxon rank sum tests comparing read-through in each sample between the two groups are indicated above the x-axis.

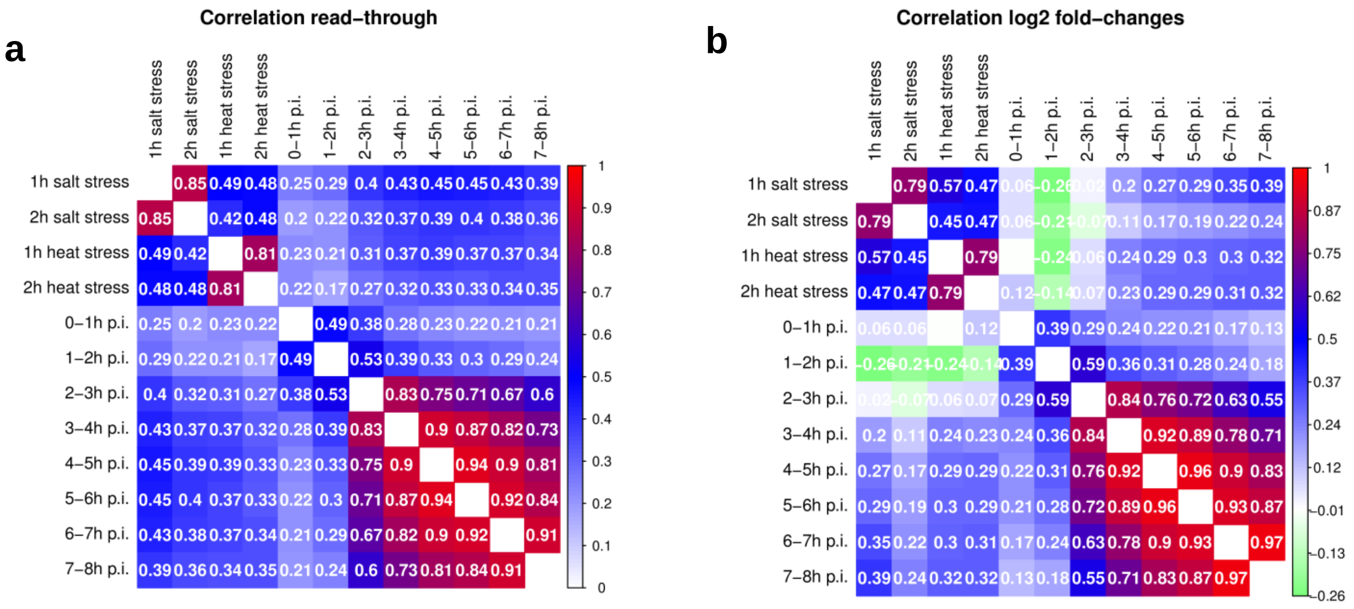


**Figure G: Correlation of read-through and log2 gene expression fold-changes**
(a) Spearman correlation for read-through values between all samples for genes with gene expression fold-changes <2 in all samples. (b) Spearman correlation for log2 gene expression fold-changes (relative to uninfected/unstressed cells) between all samples.

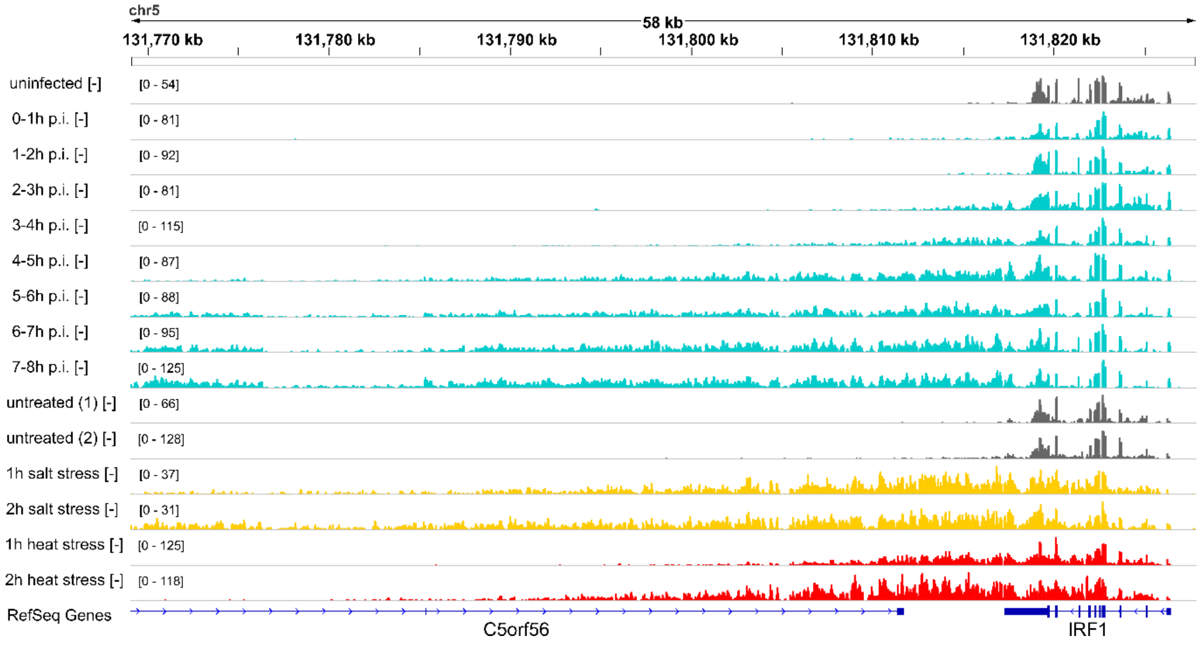


**Figure H: IRF1 read-through**

4sU-seq read coverage for the IRF1 gene in uninfected/untreated samples (gray), during HSV-1 infection (cyan) and in salt (yellow) and heat (red) stress. Only reads mapping to the corresponding strand are shown. Read coverage ranges and RefSeq gene annotation are indicated as described in Figure A(a,b). Extensive DoTT/DoG transcription is observed in all conditions.

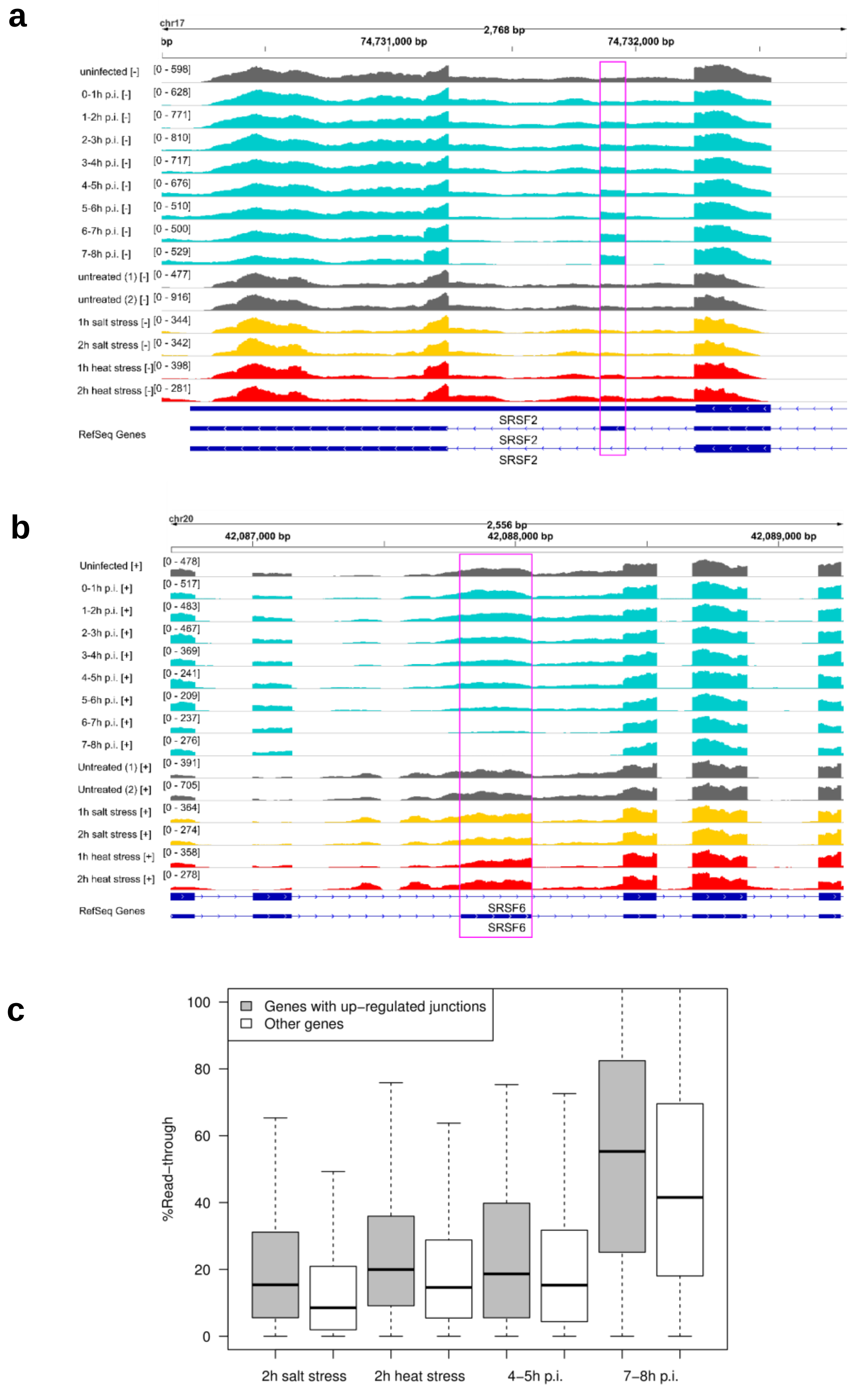


**Figure I: Induced aberrant splicing associated with DoTT/DoG transcription**

(a-b) Induced NMD-associated splicing events that are either only observed in HSV-1 infection (SRSF2, a) or only observed in salt and heat stress (SRSF6, b). 4sU-seq read coverage is shown for uninfected/untreated samples (gray), during HSV-1 infection (cyan) and in salt (yellow) and heat (red) stress. Only reads mapping to the corresponding strand are included in the Figure. Read coverage ranges and RefSeq gene annotation are indicated as described in Figure A(a,b). The induced alternative splicing events are highlighted by pink rectangles. (c) Boxplots indicating the distribution of read-through for genes with (gray) and without (white) upregulated intragenic splicing events for 2h salt or heat stress and 4-5h p.i. and 7-8h p.i. HSV-1 infection.

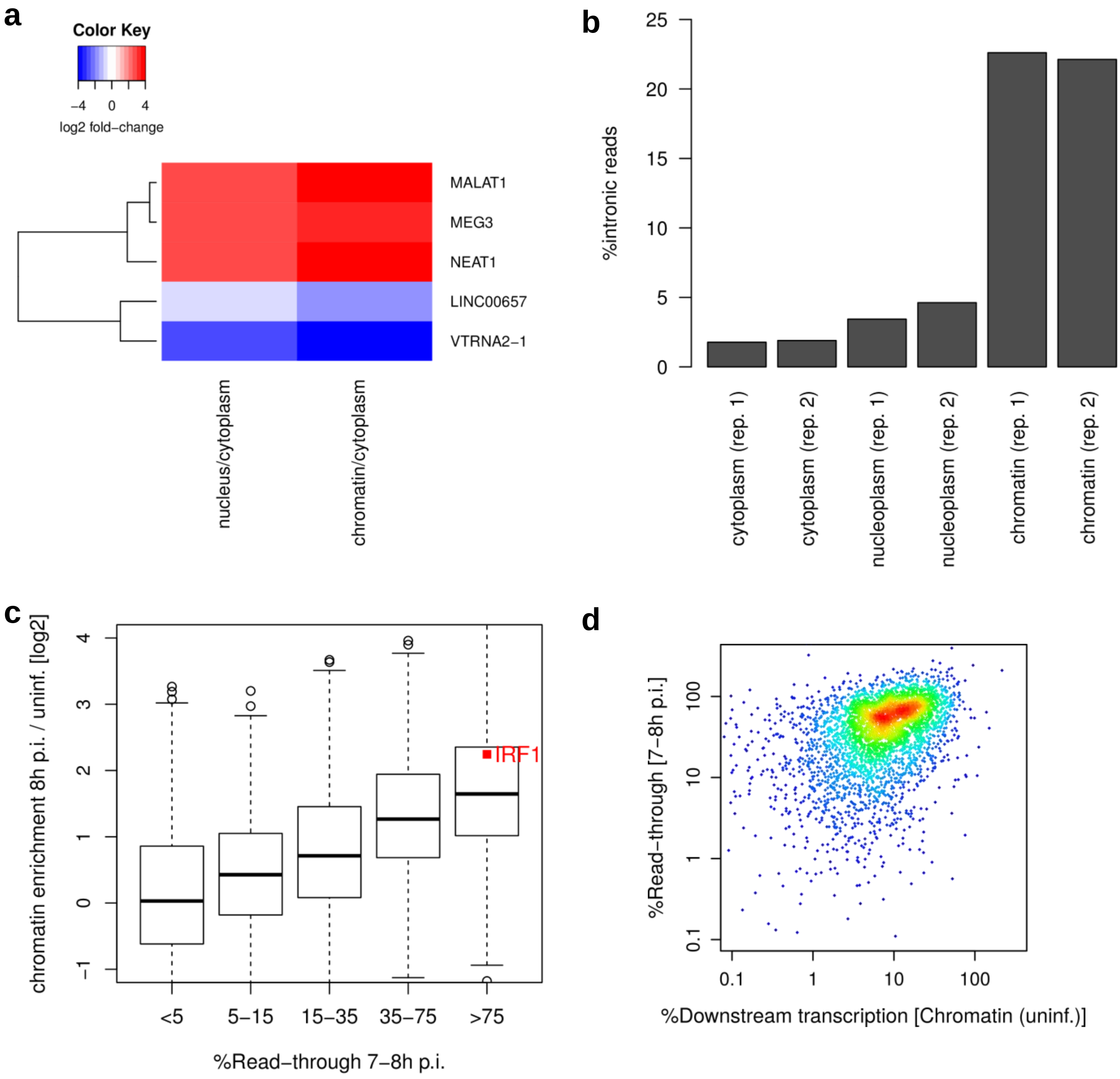


**Figure J: Separation of cytoplasmic, nucleoplasmic and chromatin-associated RNA and correlation to read-through**

(a) log2 gene expression (FPKM) fold-changes for nucleoplasmic vs. cytoplasmic RNA and chromatin-associated vs. cytoplasmic RNA for three well-described nuclear lincRNAs (MALAT1, NEAT1, MEG3) and two cytoplasmic lincRNAs (LINC00657, VTRNA2-1).

(b) Percentage of intronic reads (= 100 x no. intronic reads / ( no. intronic reads + no. exonic reads)) for cytoplasmic, nucleoplasmic and chromatin-associated RNA shows an enrichment of intronic reads in chromatin-associated RNA. (c) Boxplots indicating the distribution of log2 ratios of chromatin enrichment (= gene FPKM in chromatin-associated RNA/gene FPKM in cytoplasmic RNA) at 8h p.i. compared to uninfected cells. Ratios are shown separately for groups of genes with different amounts of read-through in 7-8h p.i. 4sU-RNA. The value for IRF1 is highlighted in red. (d) Scatterplot of read-through at 7-8h p.i. against the percentage of transcription downstream of genes identified in chromatin-associated RNA of uninfected/untreated cells. Colors indicate density of points (red=highest density, blue=lowest density).

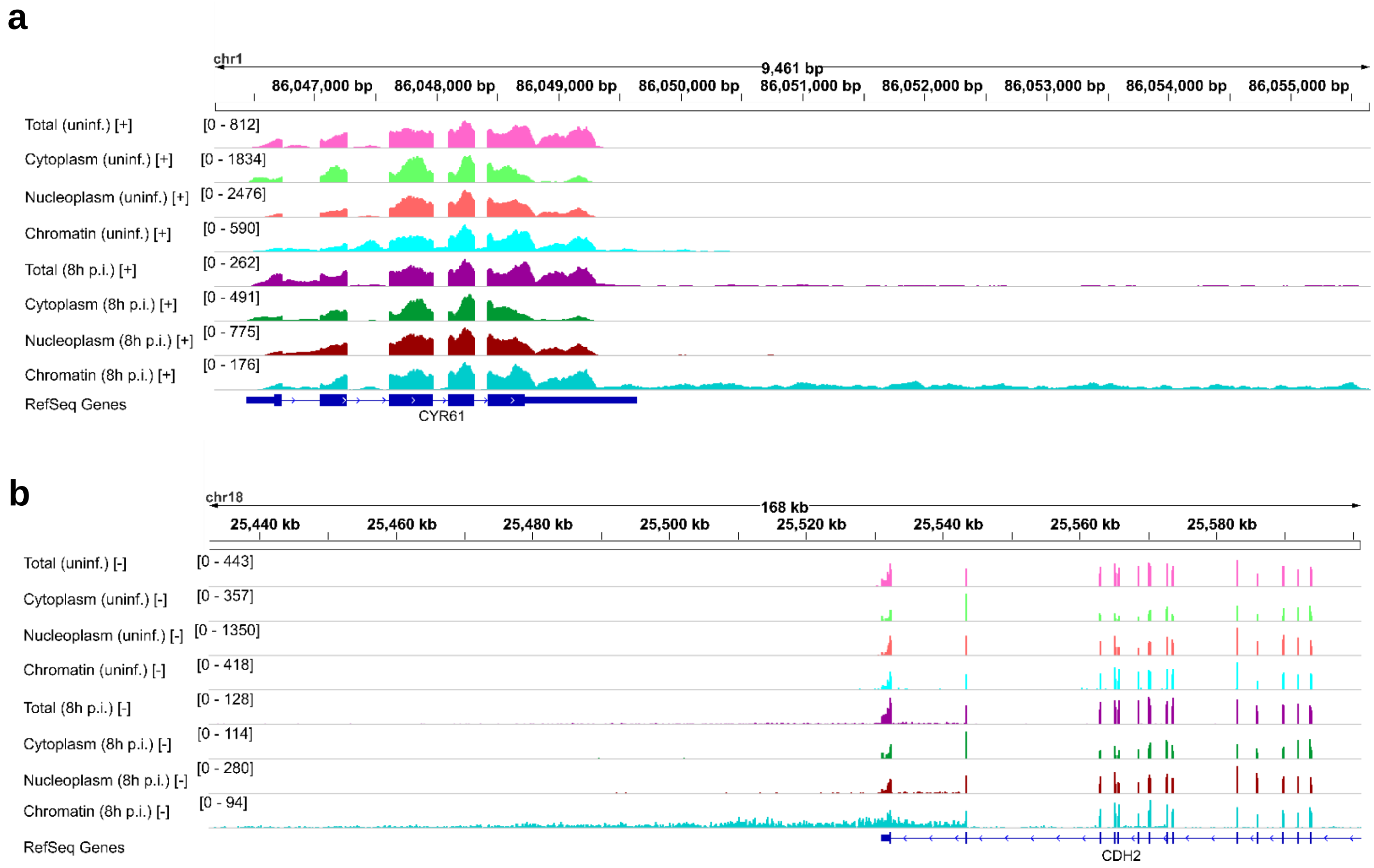


**Figure K: Subcellular localization for read-through transcripts not released from the chromatin**

(a-b) Read coverage in total (light/dark pink), cytoplasmic (light/dark green), nucleoplasmic (light/dark red) and chromatin-associated RNA (light/dark cyan) in uninfected cells (light colors) and at 8h p.i. (dark colors) for the genes CYR61 (a) and CDH2 (b). Only reads mapping to the corresponding strand are included in the Figure. Read coverage ranges and RefSeq gene annotation are indicated as described in Figure A(a,b).

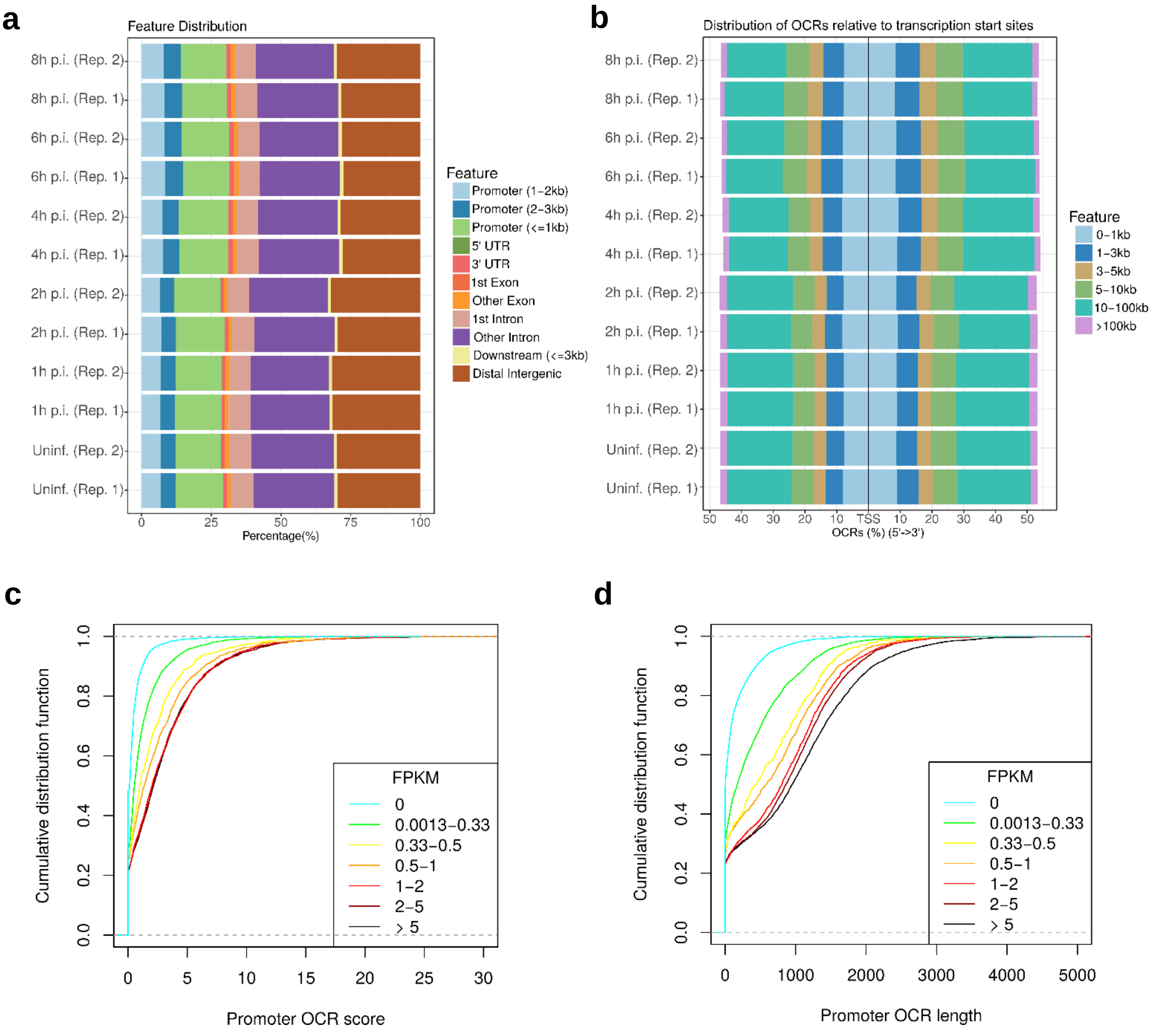


**Figure L: ATAC-seq identifies open chromatin predominantly around promoters of expressed genes**

(a) Distribution of identified open chromatin regions (OCRs) relative to genomic features shows an enrichment at promoters and gene regions. (b) Distribution of OCRs relative to transcription start sites. Assignment of OCRs to genes was performed using ChIPseeker. (c-d) Empirical cumulative distribution functions of (c) promoter OCR score as calculated by F-Seq and (d) OCR length for protein-coding genes with different expression levels in uninfected cells. The empirical cumulative distribution function indicates the fraction of genes with at most the OCR score or length indicated at this point on the x-axis. Assignment of OCRs to gene promoters was performed using ChIPseeker and OCR scores and lengths were averaged between replicates. If no promoter OCR was identified for a gene, OCR score and length were set to 0.

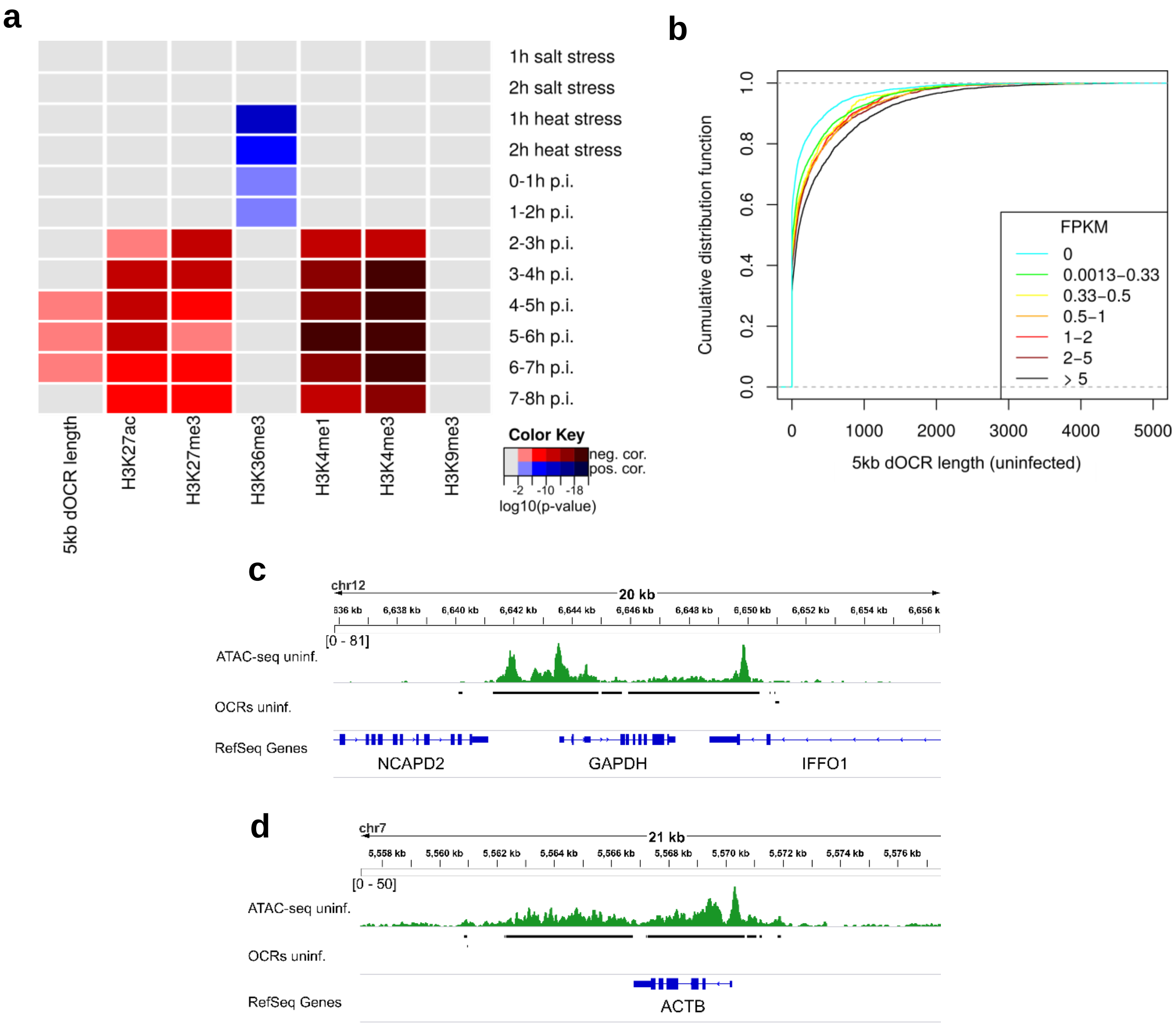


**Figure M: Chromatin accessibility and presence of histone markers downstream of genes in uninfected/unstressed cells**

(a) Presence of open chromatin downstream of genes was evaluated by determining the number of nucleotides in the first 5kb downstream of gene 3’ ends that overlapped an OCR (denoted as 5kb dOCR length). Similarly, presence of histone marks downstream of genes (=downstream histone mark length) was evaluated by determining the number of nucleotides in the first 5kb downstream of gene 3’ ends that overlap ChIP-seq narrow peaks for the corresponding histone marks (obtained from ENCODE for HFF). We then calculated the Spearman rank correlation between read-through in each condition and 5kb dOCR length or downstream histone mark length in uninfected/unstressed cells, respectively. Significance of correlations was calculated using the cor.test function in R. FDR adjusted p-values for all samples are indicated by colors (red for negative correlations, blue for positive correlations. (b) Empirical cumulative distribution functions indicating the fraction of genes (y-axis) with at most a certain 5kb dOCR length in uninfected/unstressed cells (x-axis). Genes were grouped according to FPKM in uninfected cells. (c,d) ATAC-seq read coverage (green, no strand specificity) and identified OCRs (from replicate 1, black lines) in uninfected cells for the highly expressed genes GAPDH (c) and ACTB (d). This shows extensive OCRs across the whole gene body that extend into the downstream intergenic regions.

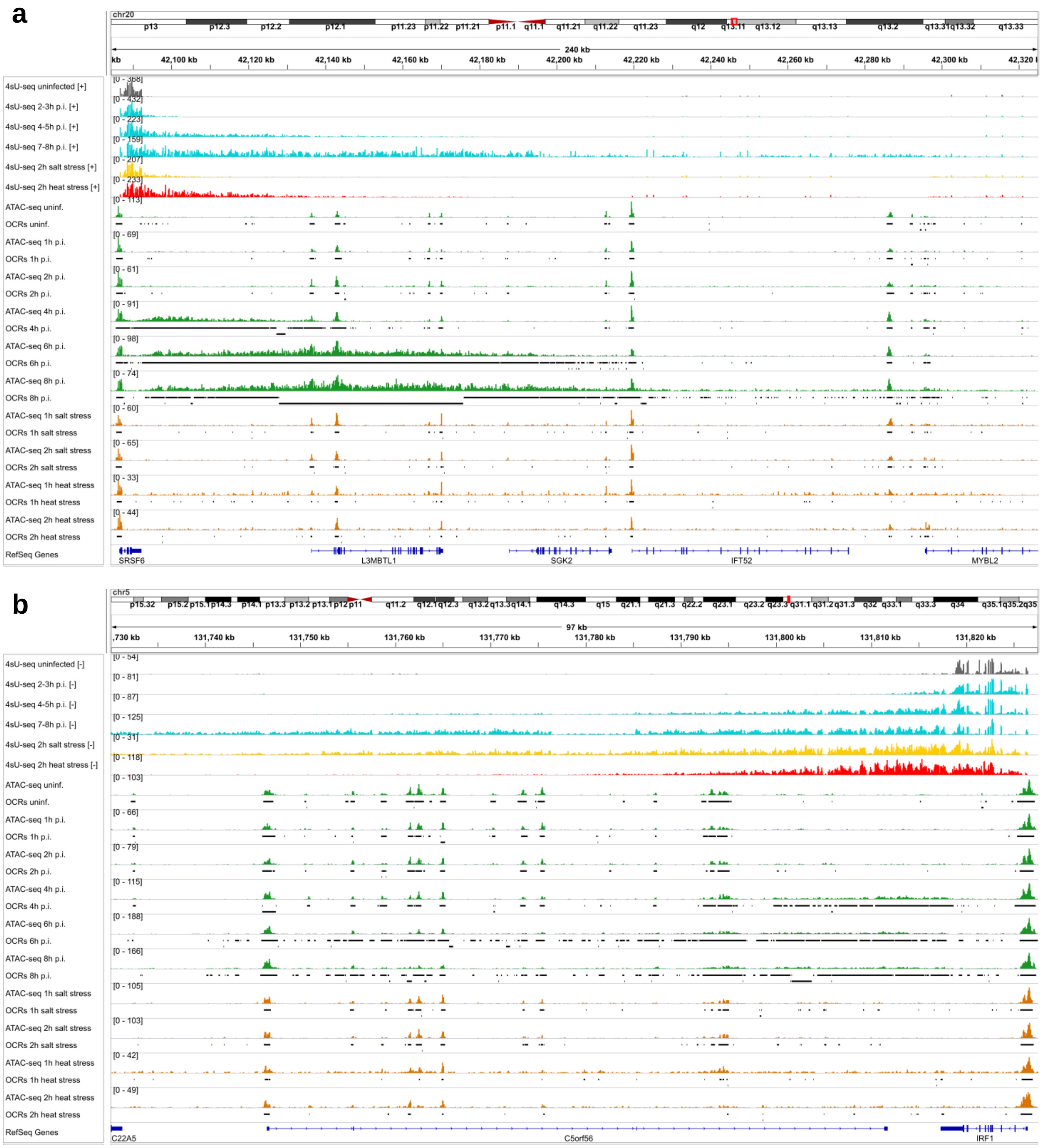


**Figure N: Open chromatin downstream of individual genes during HSV-1 infection**

4sU-seq read coverage for the genes SRSF6 (a) and IRF1(b) (strand-specific, grey = uninfected/unstressed, cyan = selected time-points of HSV-1 infection, yellow = 2h salt stress, red = 2h heat stress) as well as ATAC-seq data (no strand specificity, green = HSV-1 infection, brown = salt and heat stress) and identified open chromatin regions (OCRs, black lines). The OCRs shown here were derived from replicate 1. Read coverage ranges and RefSeq gene annotation are indicated as described in Figure  A(a,b).

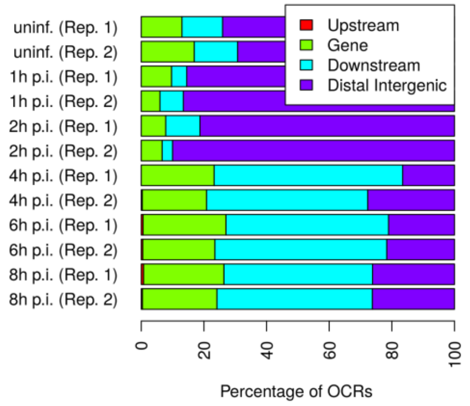


**Figure O: Enrichment of long OCRs downstream of genes during HSV-1 infection**

Distribution of long (≥5kb) OCRs relative to genes before and during HSV-1 infection (Downstream = OCR overlapping 3kb downstream of gene 3'end; Gene = OCR overlapping gene; Upstream = OCR overlapping 3kb upstream of gene 5'end; Distal intergenic = neither overlapping downstream, gene, or upstream region.)

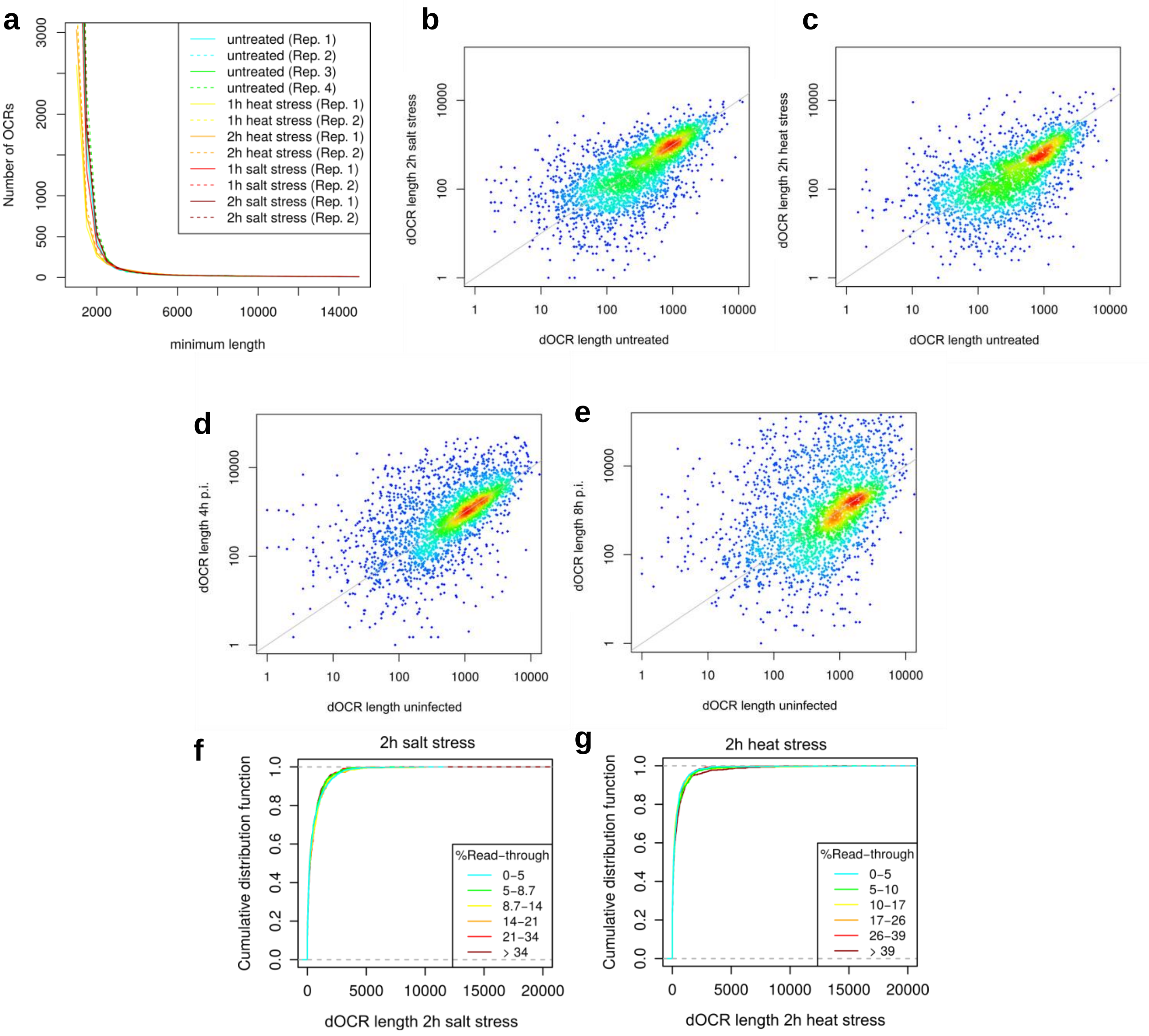


**Figure P: Chromatin accessibility downstream of genes affected by DoG transcription in salt and heat stress**

(a) Numbers of identified OCRs (y-axis) with a minimum length (x-axis) for all ATAC-seq samples from the salt and heat stress experiment. Results for replicates are shown separately. (b-e) Scatterplots comparing dOCR length in untreated (b-c) or uninfected (d-e) cells against dOCR length after 2h salt stress (b), 2h heat stress (c), 4h (d) and 8h (e) HSV-1 infection. Colors indicate density of points (red = highest density, blue = lowest density). The grey line indicates equal values. For salt and heat stress no increase beyond random fluctuations is observed, while at 4h of HSV-1 infection or later, dOCR length increases considerable for a large number of genes. (f-g) Empirical cumulative distribution functions indicating the fraction of genes (y-axis) with at most a certain dOCR length (average between two replicates) at (f) 2h salt stress or (g) 2h heat stress (x-axis). Genes were grouped according to read-through in either (f) 2h salt stress or (g) 2h heat stress. For grouping, genes with ≤5% read-through were assigned to the first group and the remaining genes were divided in equal-sized groups according to read-through.

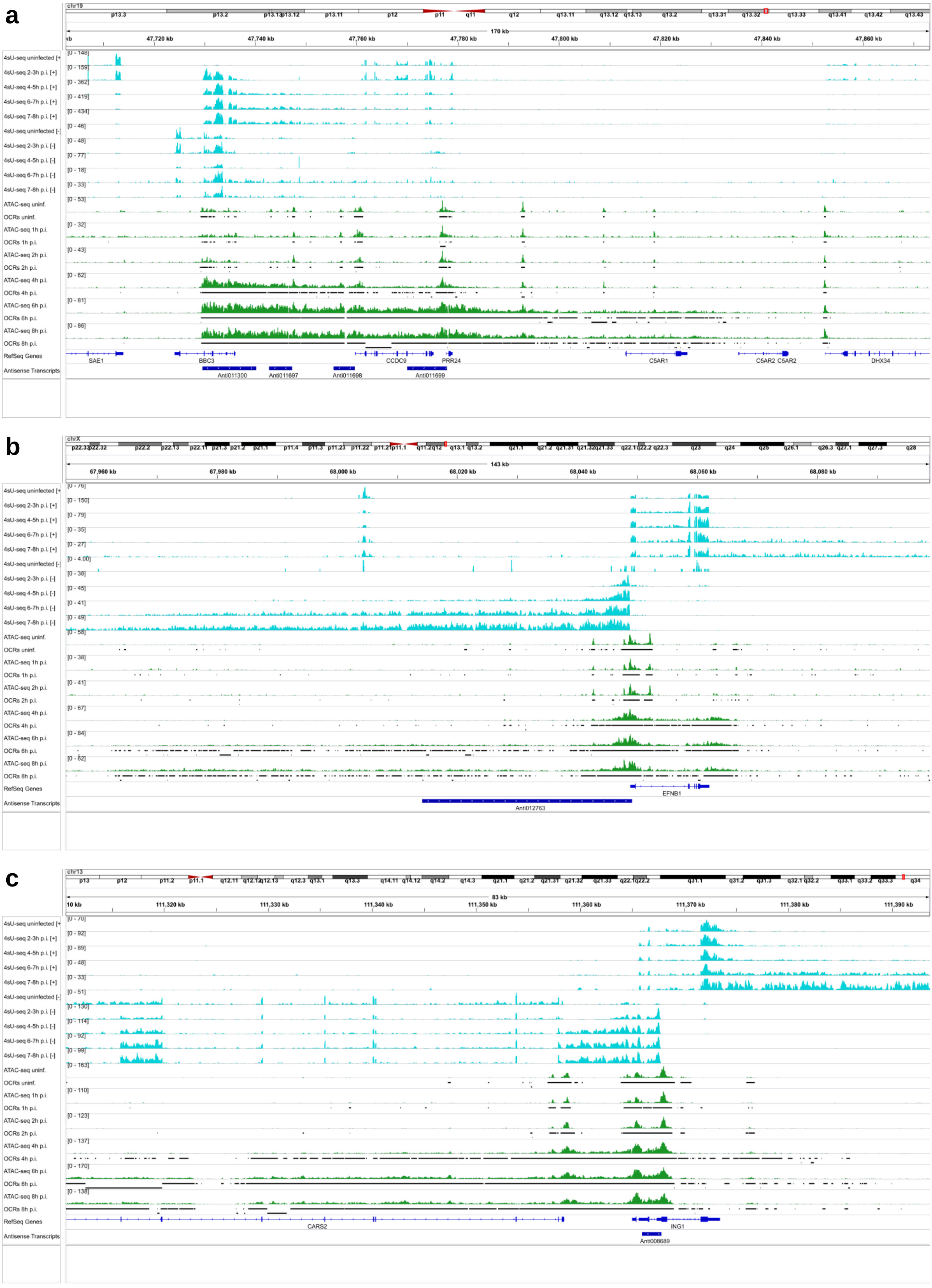


**Figure Q: Legend follows**

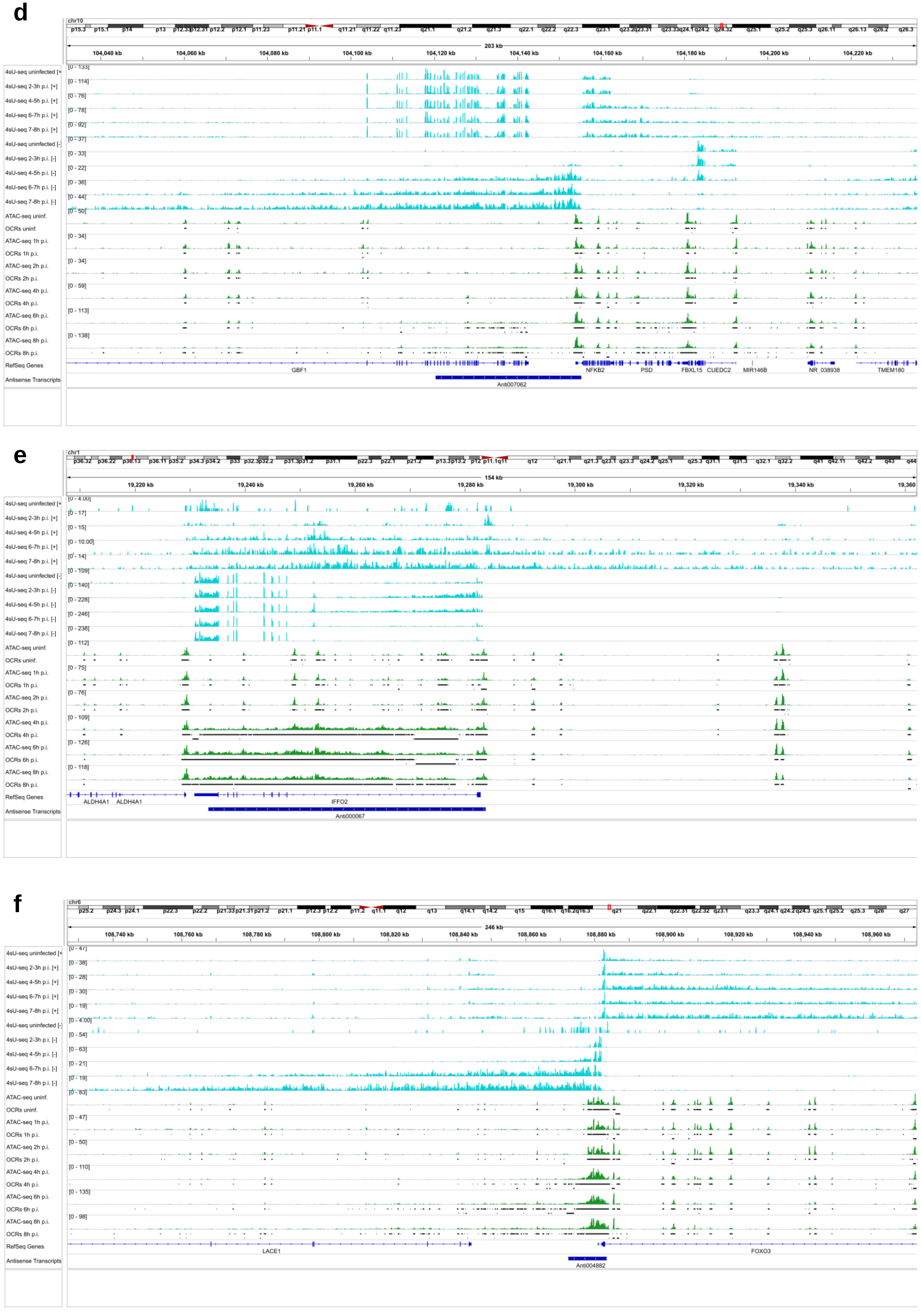


**Figure Q: Legend follows**

g

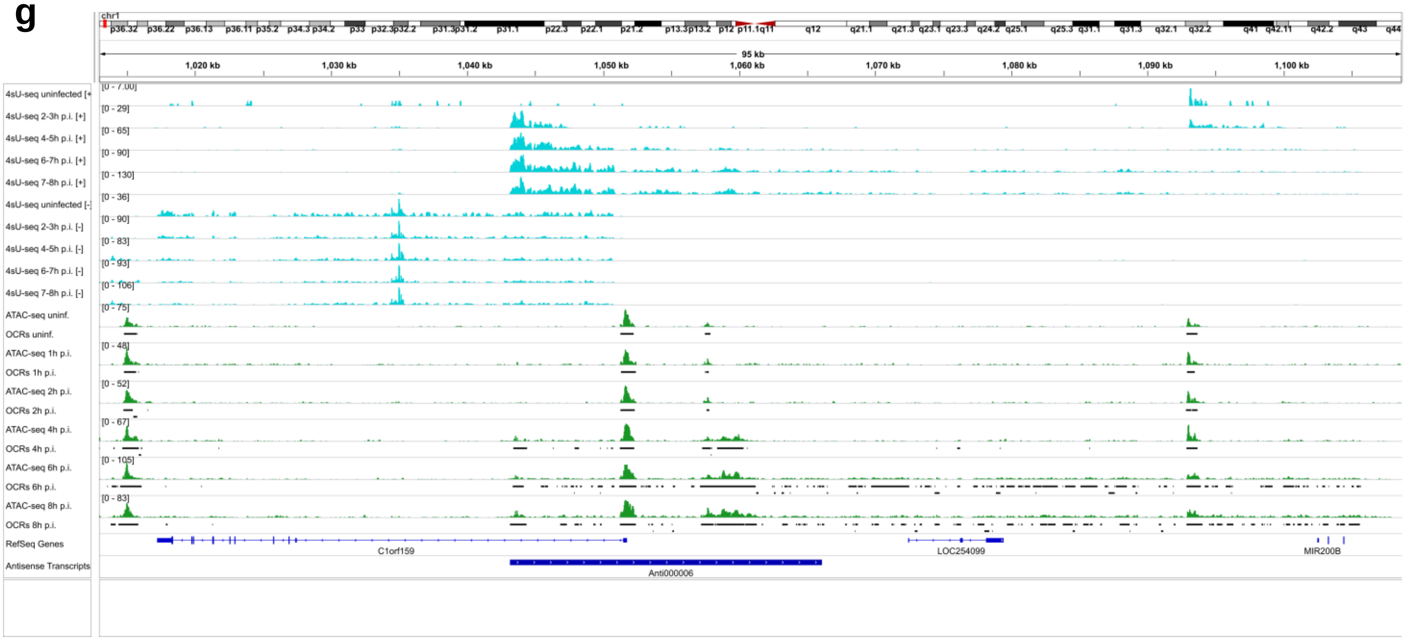


h

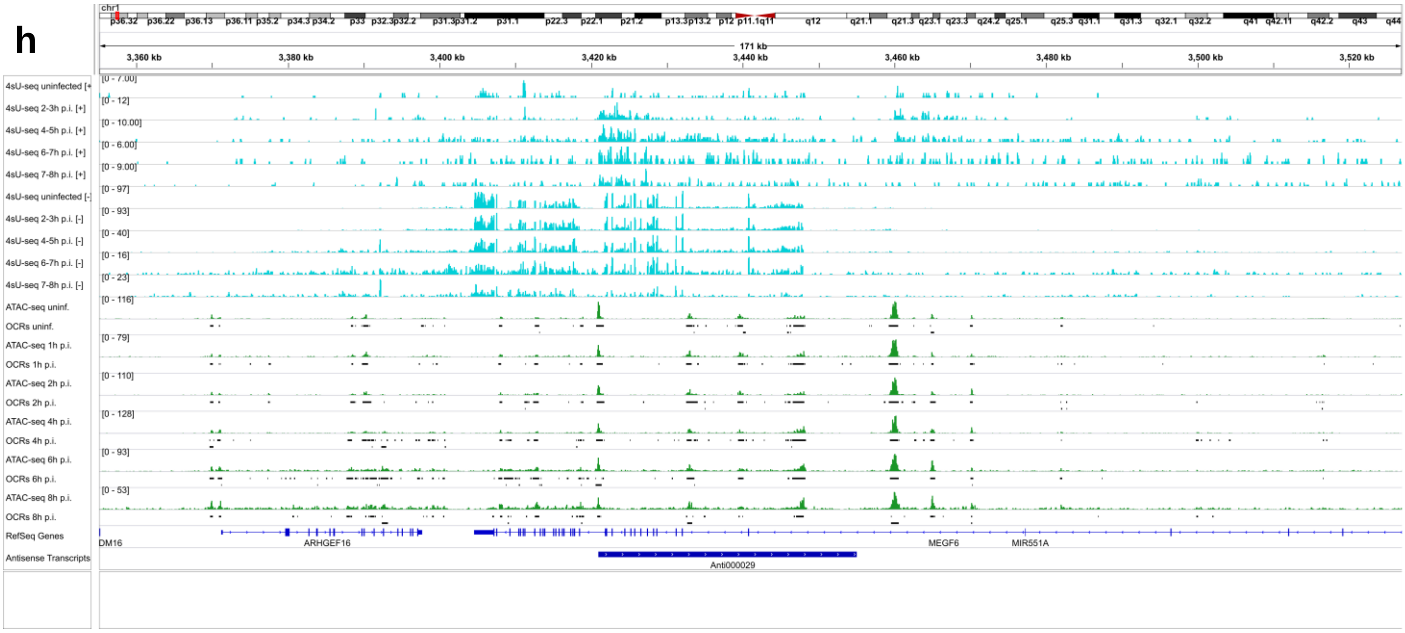


i

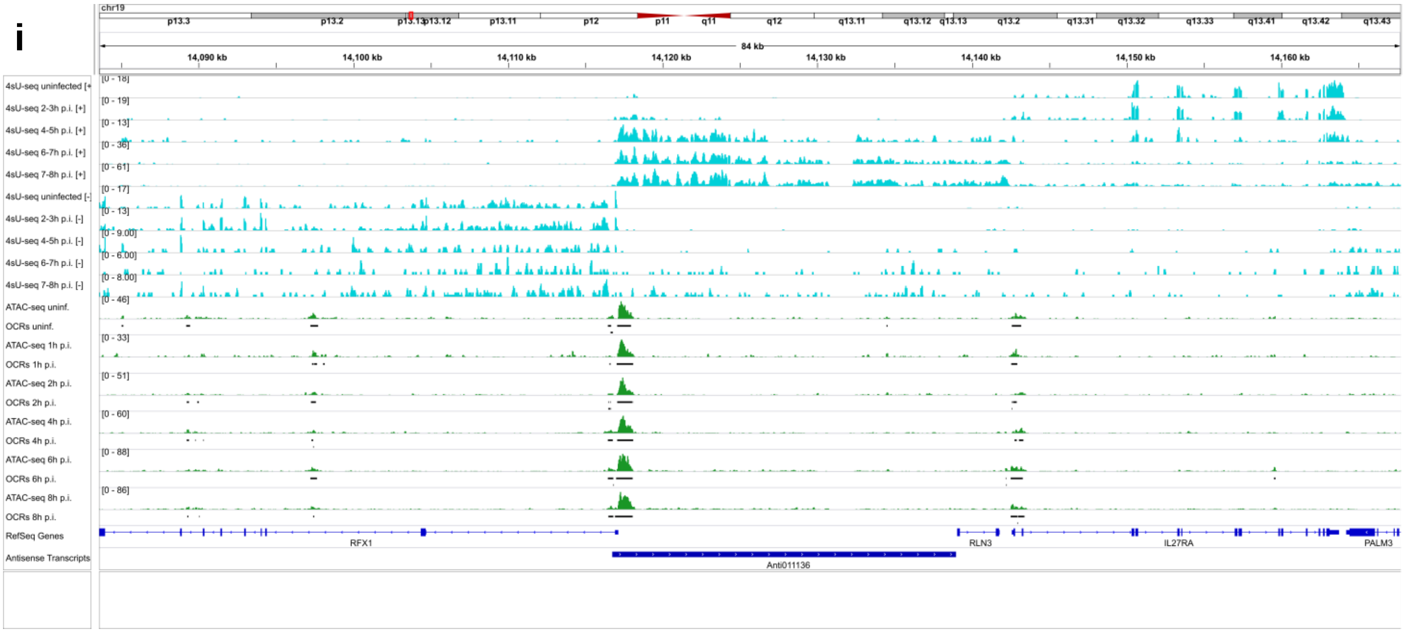


**Figure Q: Legend follows**

j

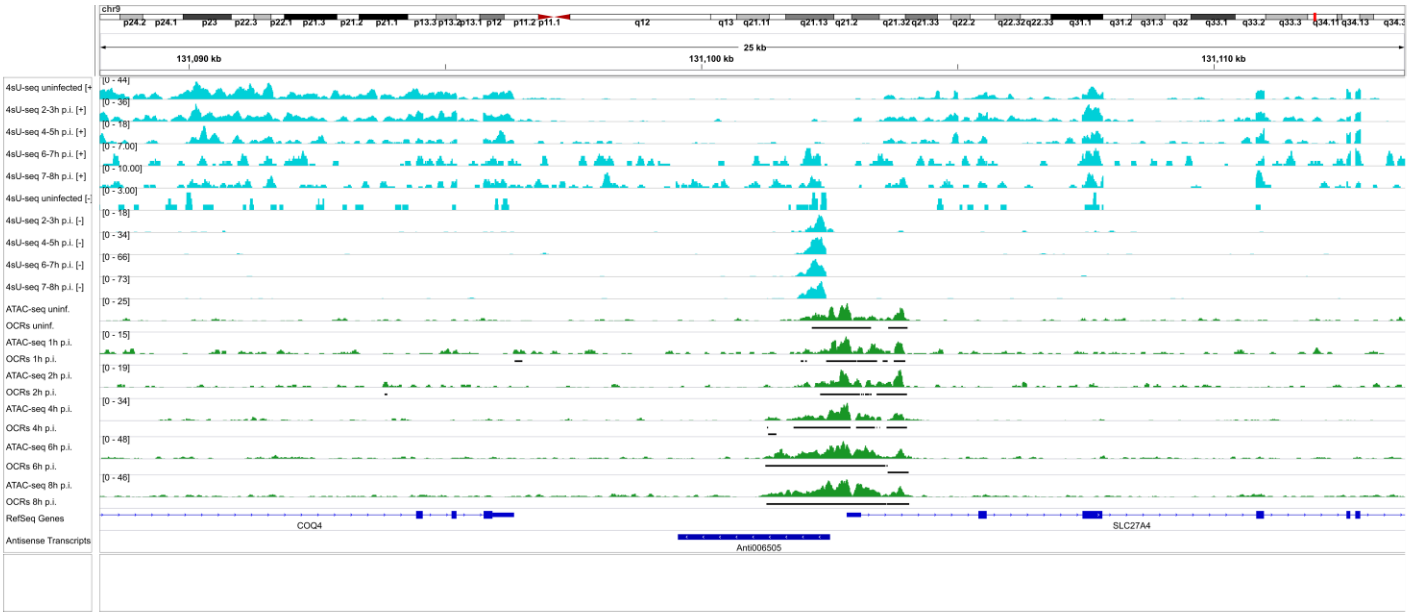


k

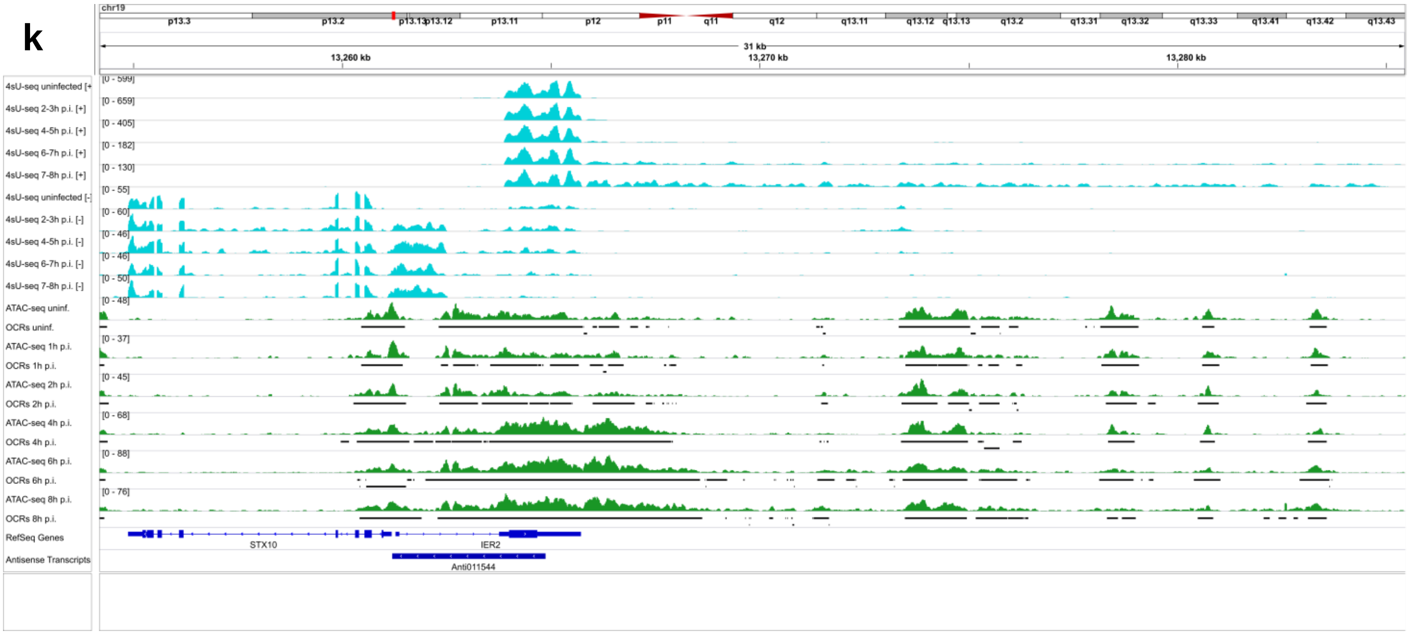


**Figure Q: Open chromatin around antisense transcripts during HSV-1 infection**
4sU-seq read coverage and open chromatin regions for 11 antisense transcripts identified by Wyler et al. and confirmed by RT-qPCR and Nanostring nCounter assays. 4sU-seq read coverage is shown for uninfected cells and at selected time-points during infection (cyan, strand-specific). Furthermore, ATAC-seq read coverage (green, no strand specificity) and identified open chromatin regions (OCRs, black lines) are shown for uninfected cells and at 1, 2, 4, 6, 8 h p.i. The OCRs shown here were derived from replicate 1. Read coverage ranges and RefSeq gene annotation are indicated as described in Figure A(a,b). The bottom line indicates the region and strand (direction of arrowheads) of the antisense transcripts identified by Wyler et al.

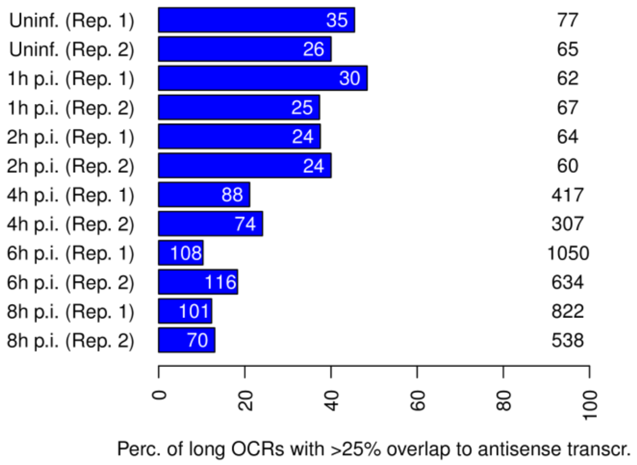


**Figure R: Overlap of OCRs with antisense transcripts**

Percentage of long (≥5kb) OCRs with at least 25% overlap to an antisense transcript from the study of Wyler et al. The total number of long OCRs (black, right) and the number of long OCRs (white, within bars) is also shown.
